# Ultra‐Weak Photon Emission as a By‐Product of Mitochondrial Oxidative Metabolism: Mechanisms, Limits, and Implications

**DOI:** 10.1002/nap2.70340

**Published:** 2026-10-22

**Authors:** Samuel Crowther, Alice Attenborough, Frank Vollmer

**Affiliations:** ^1^ Living Systems Institute, University of Exeter Exeter UK

**Keywords:** biophotons, mitochondria, oxidative phosphorylation, oxidative stress, reactive oxygen species, single‐photon detection, ultra‐weak photon emission

## Abstract

Ultra‐weak photon emission (UPE) is spontaneous, low‐intensity light produced during aerobic metabolism. UPE has multiple cellular sources; we focus on the mitochondrial contribution, which arises predominantly from reactive oxygen species (ROS) chemistry and electronically excited products, linking UPE to redox state and mitochondrial function. We treat UPE as a by‐product of oxidative metabolism, not as a dedicated signalling channel. We outline mitochondrial oxidative pathways generating triplet excited carbonyls and singlet oxygen, and distinguish baseline UPE from stimulated emission under oxidative stress, lipid peroxidation and cell death. An energy budget for the mitochondrial electron transport chain shows that no single transition can emit a visible or near‐infrared photon: the largest step corresponds to ∼2180 nm, and the residual after proton‐pumping to ∼3000 nm, where the sample's thermal emission dominates. Measured UPE, at rest and under stress, therefore reflects ROS‐driven chemiexcitation, and the two regimes differ chiefly in flux. We review detectors, from photomultiplier tubes and low‐light cameras to single‐photon avalanche diodes and emerging cryogenic and nanophotonic sensors, and organise applications across medicine, neuroscience, agriculture, food science, dermatology and fundamental biology within a two‐regime framework (spontaneous vs. stimulated). We conclude with experimental priorities for establishing UPE as a quantitative reporter of oxidative metabolism.

## Graphical abstract

Mitochondrial oxidative metabolism generates ROS. Downstream chemiexcitation triplet carbonyls, singlet oxygen; produces ultra‐weak photon emission (UPE). Energetic bounds restrict well‐coupled respiration to extremely weak, red/NIR‐biased emission. Stronger visible UPE indicates elevated oxidative flux, linking measured photon counts to redox state and mitochondrial function.

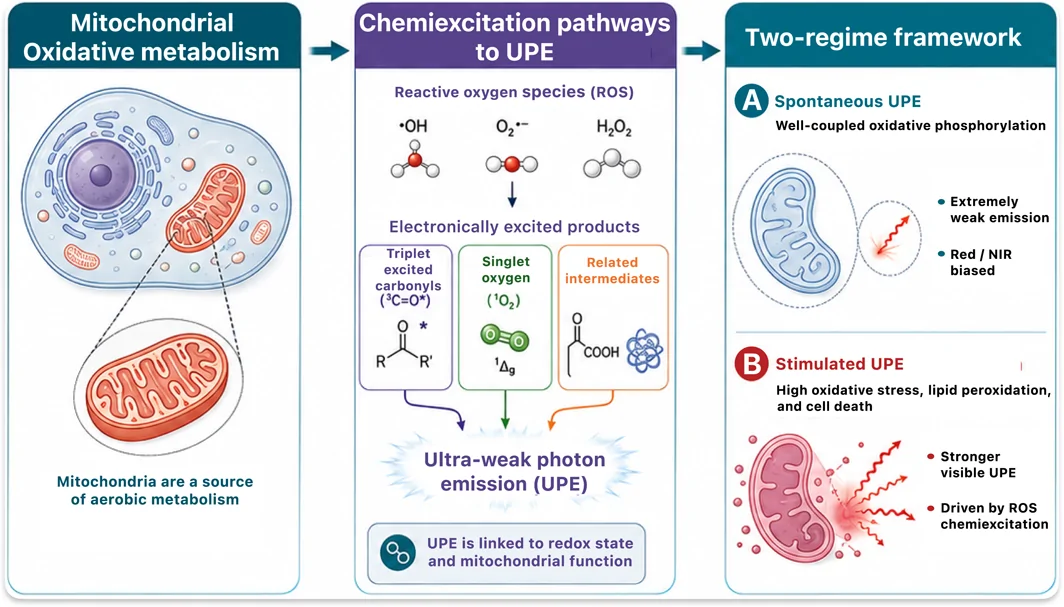

## 1 Introduction

### 1.1 UPE and the Biophoton Problem

Biological systems at nonzero temperature are never truly dark. Under aerobic conditions, oxidative metabolism and associated radical chemistry inevitably generate small populations of electronically excited intermediates that can decay radiatively, giving rise to ultra‐weak photon emission (UPE) in the visible and near‐infrared (NIR) range [1, 2, 3, 4]. This emission is many orders of magnitude weaker than conventional bioluminescence or fluorescence: typical reported intensities from cells or tissues lie around $10^{-1}$‐$10^{2}$ photons s^−1^ cm^−2^, often requiring long integration times and highly optimised detectors (Figure 1).

**FIGURE 1:**
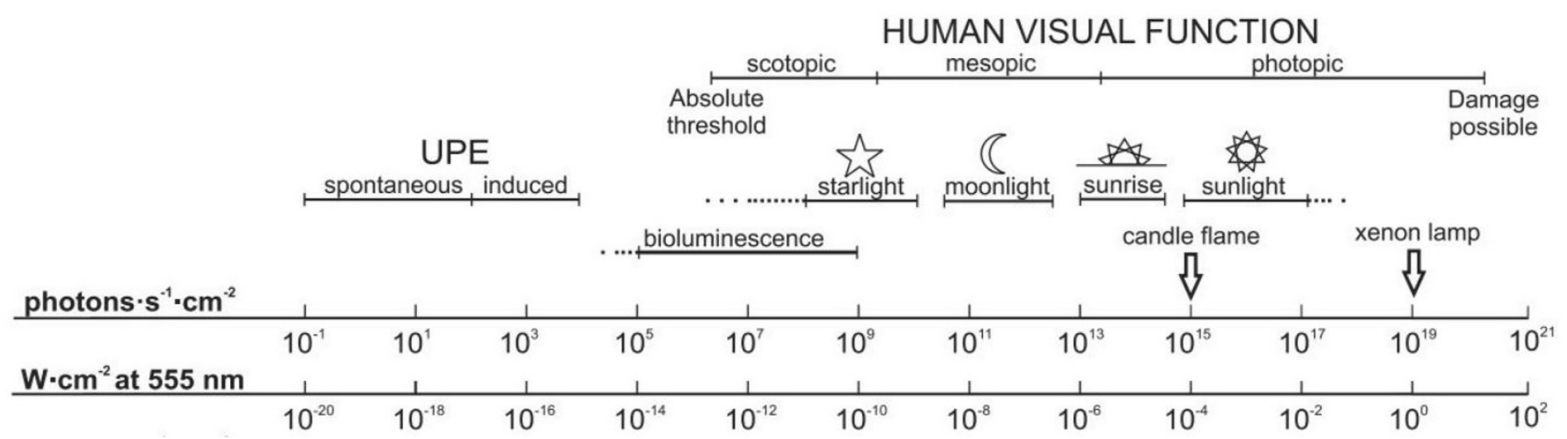
Intensity ranges of various photon emission compared to the sensitivity of the human eye. Reprinted from M. Cifra and P. Pospíšil, ‘Ultra‐weak photon emission from biological samples: Definition, mechanisms, properties, detection and applications’, *Journal of Photochemistry and Photobiology B: Biology*, vol. 139, pp. 2–10, Copyright (2014), with permission from Elsevier [4].

Since the earliest quantitative measurements in the 1960s–1970s, the observation of such emission has given rise to two rather different narratives. In one, UPE is treated as a chemically understandable by‐product of oxidative stress, lipid peroxidation and delayed relaxation of photochemically primed states [3, 5]. In the other, it is elevated to a quasi‐functional entity under the label ‘biophotons’: inherently coherent, sometimes laser‐like photons purportedly involved in long‐range cellular communication, morphogenesis or even quantum information processing in the brain [4, 6, 7, 8]. These two views are often discussed together, despite relying on very different implicit assumptions about how many photons are actually available, at what wavelengths and under what physiological conditions.

Although this review focuses on the mitochondrial contribution to UPE, mitochondria are not the only cellular source of the underlying reactive chemistry. Reactive oxygen species (ROS) are also generated at the endoplasmic reticulum and peroxisomes, by NADPH oxidases and other oxidoreductases, and in plants by chloroplasts and apoplastic enzymes; their relative contributions are cell type dependent. Mitochondria also produce ROS; however, whether they are the dominant source of ROS remains debated; measurements indicate that mitochondria are a significant but not necessarily the largest source of cellular ROS [9, 10]. The bulk UPE measured from intact cells or tissues therefore reflects total oxidative activity rather than mitochondrial metabolism alone. We concentrate on the mitochondrial electron transport chain (ETC) as the most energetically tractable source to bound quantitatively (Section 3) and the one to which direct ‘biophoton’ emission is most often attributed; the limits we derive apply to that mitochondrial contribution specifically, not to the whole cellular signal.

The issue addressed in this review is the realistic UPE emission expected from mitochondrial sources. We ask three questions: how much radiative loss the mitochondrial ETC and associated ROS chemistry can support under well‐coupled, near‐homeostatic conditions; what spectral distributions follow from the underlying energetics, and how they compare with reported ‘biophoton’ spectra; and under what circumstances experimentally accessible UPE signals correspond to spontaneous metabolism rather than strongly stimulated, high‐ROS, high‐damage chemistry.

Addressing these questions requires bringing together three strands of evidence that have often been considered separately: (i) the thermodynamics and stoichiometry of oxidative phosphorylation (Section 3); (ii) the specific ROS and chemiexcitation pathways that generate excited products and photons (Section 2); and (iii) the practical limits and artefacts of modern single‐photon detectors (Section 4). On that basis, we propose a simple two‐regime framework: a spontaneous regime in which UPE is an extremely weak by‐product of basal ROS chemistry in well‐coupled oxidative metabolism, and a stimulated regime in which elevated ROS flux drives the same chemistry to a much higher intensity, from physiological activation through to membrane damage and cell death.

### 1.2 Historical Discovery and Terminology

The idea that living systems might emit weak radiation dates back at least to the early twentieth century. In the 1920s, Gurwitsch reported that onion root tips appeared to influence mitosis in neighbouring roots through a putative ‘mitogenetic radiation’, which he attributed to ultraviolet (UV) photons in the 200–300 nm range [13]. These experiments, which used quartz windows and photographic plates, sparked considerable interest and controversy. Although some contemporaries claimed partial replication, many others failed to reproduce the effect, and methodological criticisms (e.g., inadequate shielding, uncontrolled contamination, subjective plate reading) eventually led to broad scepticism about mitogenetic radiation as a robust phenomenon.

The modern era of UPE research began in the 1950s–1960s with the development of photomultiplier tubes (PMTs) and other highly sensitive photon detectors. Russian and Japanese groups, among others, demonstrated that a wide variety of biological samples including plant leaves, seeds, bacterial and eukaryotic cell suspensions as well as animal tissues emit photons in the visible range at ultra‐low intensities [5, 14]. This work established that UPE is a ubiquitous feature of aerobic life, tightly linked to oxidative metabolism and stress. Experiments also revealed that emission depends strongly on environmental conditions: it increases with metabolic activity, UV exposure and chemical oxidants, and decreases with antioxidants, quenchers or transitions to anaerobic conditions [1, 3].

In the 1970s, Popp and coworkers introduced the term ‘biophotons’ and advanced a more radical interpretation, arguing on the basis of photon counting statistics and coherence measurements that UPE might exhibit laser‐like properties and play a role in cellular communication and morphogenesis [6, 7]. They proposed that DNA or other ordered biomolecules could act as sources and storage media for coherent photons, and that biological systems might use these photons as a regulatory network. These ideas stimulated extensive theoretical and experimental work, but also attracted criticism regarding the interpretation of coherence measurements, the neglect of mundane chemiluminescent mechanisms, and the absence of clear, reproducible biological functions attributable to such weak signals [4, 5].

Over the following decades, the field diversified. With mechanistic studies in biochemistry and photobiology clarified how ROS, lipid peroxidation and delayed luminescence (DL) from pigments and proteins produce broad, weak emission bands [1, 3, 15]. Accompanied by application oriented work explored UPE as a noninvasive marker of oxidative stress in medicine, agriculture, food science and dermatology [14, 15, 16, 17, 18, 19, 20, 21]. As the field grow so did the list of terminology: ‘ultra‐weak chemiluminescence’, ‘delayed luminescence’, ‘biophoton emission’ and ‘ultra‐weak photon emission’ are often used interchangeably, even when referring to distinct physical mechanisms and regimes. See Table 1 for a full list of terminology.

**TABLE 1:** Accepted terms referring to biological autoluminescence (BAL), often reported under the umbrella term ‘UPE’. BAL can be ultra‐weak in bulk but not necessarily in all microenvironments; ‘UPE’ is therefore a regime descriptor rather than a mechanistic definition (Figure 1) [4, 11, 12].

Terminology
Autoluminescence
Weak luminescence
Biological autoluminescence (BAL)
Biophoton/biophoton emission
Low‐level chemiluminescence
Spontaneous chemiluminescence
Spontaneous ultra‐weak light emission
Ultra‐weak bioluminescence
Ultra‐weak photon emission (UPE)

In this review, we use UPE as an umbrella term for spontaneous, ultra‐low‐intensity photon emission from living matter in the absence of externally added luminescent substrates, while distinguishing between spontaneous and stimulated regimes. We reserve ‘biophoton’ for historical discussion and for hypotheses that propose functional roles beyond those justified by energetics and known chemistry. The mechanistic basis for UPE rooted in mitochondrial ROS production, lipid and protein oxidation and excited intermediates such as triplet carbonyls and ^1^O_2_ is reviewed in Section 2. Section 5 revisits some of the claims of the original ‘biophoton’ literature in the light of this mechanistic and quantitative framework.

### 1.3 Spontaneous Versus. Stimulated UPE: A Two‐Regime View

A core theme of this article is that not all UPE is created equal. The same underlying oxidative pathways can operate at very different fluxes and in very different physiological contexts, leading to qualitatively distinct emission regimes. For the purposes of interpretation, it is useful to distinguish between the following:

- A spontaneous regime, mitochondria are well coupled, ROS fluxes are low, antioxidant systems are effective, and UPE is a by‐product of routine oxidative phosphorylation.
- A stimulated regime, strong perturbations drive large ROS bursts, lipid peroxidation cascades, membrane damage and cell death, producing emission orders of magnitude stronger

These regimes are separated physiologically: resting, well‐coupled respiration versus any deviation from this state. The stimulated regime is therefore inclusive of oxidative activation (e.g., exercise, immune response, circadian peaks), through to lipid peroxidation, membrane damage and cell death.

Throughout, the spontaneous regime denotes a cell at metabolic rest: baseline oxidative respiration with no fermentative shift and no externally imposed oxidative stress. This is the reference state against which all measured emission is compared. Defining it explicitly matters, as much of the confusion in the UPE literature arises from comparing signals across systems in very different metabolic states.

In the spontaneous regime, electron transport is tightly linked to adenosine triphosphate (ATP) synthesis, proton leak is modest and the redox state of the ETC fluctuates within a narrow range [2, 22]. A small but physiologically unavoidable fraction of electron flux reduces oxygen to generate superoxide that can act as signalling molecules at low flux, whereas dysregulation drives oxidative stress. Occasionally, these oxidative events produce chemiexcited intermediates (triplet carbonyls, singlet oxygen and weakly excited pigments) that relax radiatively. As discussed in Section 3.2, the fraction of respiratory free energy that can flow into such radiative channels under these conditions is extremely small, and no direct ETC transition can emit in the visible or NIR. Spontaneous UPE is therefore expected to be extremely weak, with a spectrum set by the same chemiexcited species that operate under stress.

By contrast, most of the stronger UPE signals reported in the literature involve systems that have been driven into the stimulated regime. UV or visible irradiation, exogenous oxidants, ischemia‐reperfusion, mechanical injury, intense exercise, senescence and cell death all provoke large, transient or chronic increases in ROS production [1, 5, 23, 24, 25, 26, 27, 28, 29]. These ROS cascades drive extensive lipid peroxidation and protein oxidation, populating high‐energy intermediates (dioxetanes, triplet carbonyls, ^1^O_2_) at far higher rates than in the spontaneous state. The result is UPE that can be several orders of magnitude brighter than spontaneous emission, often with prominent visible bands in the blue‐green and red regions and well‐characterised DL components [1, 3].

This two‐regime view has two immediate implications. When strong, readily detectable UPE is observed from cells or tissues using standard PMTs, charge‐coupled devices (CCDs) or electron‐multiplying CCDs (EMCCDs) over modest integration times, the system is very likely in a stimulated, high‐ROS state; UPE then reports oxidative stress, membrane damage and repair rather than the subtle fluctuations of normal mitochondrial function. Conversely, any attempt to assign functional signalling roles to spontaneous UPE must contend with the severe photon budget and spectral constraints of well‐coupled oxidative phosphorylation (Section 3)

In this review, fast‐transient UPE refers to short‐lived elevations in photon emission above baseline that occur on sub‐second to minute timescales. Importantly, the observed width/shape of a ‘transient’ is dependent on (burst may appear as a spike or a broadened pulse) detector integration time (bin width) and any smoothing/detrending applied. Fast transients are commonly reported during abrupt redox perturbations or oxidative bursts (e.g., plant hypersensitive responses and acute oxidative challenges), whereas slower components (tens of minutes to hours) more often reflect sustained shifts in oxidative metabolism and/or longer lived oxidation products. For detector and analysis specific considerations, see Section 4.3 [4, 14, 16, 30].

### 1.4 Circadian Regulation, Redox Tone and Cryptochromes

Circadian clocks impose ≈24 h structure on cellular metabolism and stress responses, including mitochondrial substrate utilisation, respiratory activity and antioxidant capacity. Because UPE is driven by oxidative chemistry (ROS production, lipid peroxidation cascades and downstream chemiexcitation), any time‐of‐day modulation of mitochondrial ROS generation or detoxification can, in principle, produce diurnal structure in measured emission even without any specialised ‘photon signalling’ function [31, 32, 33]. Consistent with this framing, redox oscillations are a conserved feature of circadian biology: peroxiredoxin oxidation cycles persist in anucleate human red blood cells and serve as a cross‐domain marker of circadian rhythmicity, underscoring that redox state itself can carry robust ≈24 h dynamics [34, 35].

Cryptochromes (CRYs) are core clock proteins with distinct roles across taxa. In insects (type I CRYs), cryptochrome acts as a flavin dependent blue light photoreceptor that couples photochemistry to clock resetting, whereas in mammals CRY1/2 function primarily as light independent transcriptional repressors within the core transcription translation feedback loop [36, 37, 38]. Importantly for a UPE review, flavin redox chemistry and radical intermediates are intrinsic to cryptochrome photochemistry in photoreceptive CRYs: light driven flavin reduction and semiquinone/radical states are well characterised in *Drosophila* CRY and related systems [39, 40]. This provides a mechanistic bridge between circadian state, flavin/redox transitions and radical chemistry [37].

Independent of any specific photoreceptor, flavin‐based radical pairs are of interest in this context because spin‐state interconversion within such pairs is coupled to the same triplet manifold that underlies chemiexcitation. The singlet–triplet branching of a radical pair determines the yield of triplet products, and it is the triplet‐state carbonyls and singlet oxygen that carry the energy capable of radiative decay in the visible. This provides a route by which the otherwise emission‐silent resting state could in principle be modulated: not through any new radiative transition, but through effects on the spin populations feeding the triplet channel. We raise this as a mechanistic connection worth further study rather than as an established source of UPE.

#### 1.4.1 Implications for UPE Experiments

Long duration UPE recordings should treat circadian phase as a potential confounder and/or signal source: report time‐of‐day, entrainment/light history, feeding state where relevant, temperature control and consider paired oxidative/redox readouts (e.g., peroxiredoxin oxidation state or antioxidant enzyme activity) when interpreting slow variations in photon emission [31, 34, 35].

The remainder of this review is organised around this two‐regime framework. Section 2 details the oxidative pathways and excited products that underlie both spontaneous and stimulated UPE, and describes how antioxidants and bystander effects modulate emission. Section 3 quantifies the energy budget of the mitochondrial ETC and derives expected photon yields and spectra under spontaneous conditions, providing a benchmark for interpreting reported ‘biophoton’ intensities. Section 4 reviews the capabilities and limitations of current photon detectors in the context of these constraints. Section 5 re‐examines medical, agricultural, food and fundamental biological applications through the lens of the two‐regime model. Finally, Section 6 synthesises these threads into a set of practical recommendations and experimental priorities for future UPE research.

## 2 Oxidative Metabolism, ROS Chemistry and UPE Generation

### 2.1 Fundamental Oxidative Pathways and Excited Products

UPE is a generic consequence of oxidative biochemistry. Whenever redox reactions generate electronically excited intermediates that decay radiatively, a small fraction of the underlying chemical free energy is released as photons. In biological systems under aerobic conditions, key contributors to such chemiexcitation include mitochondrial oxidative phosphorylation, associated ROS chemistry and downstream oxidative damage to lipids, proteins and other macromolecules (Figure 2) [2, 3, 4, 15, 41].

**FIGURE 2:**
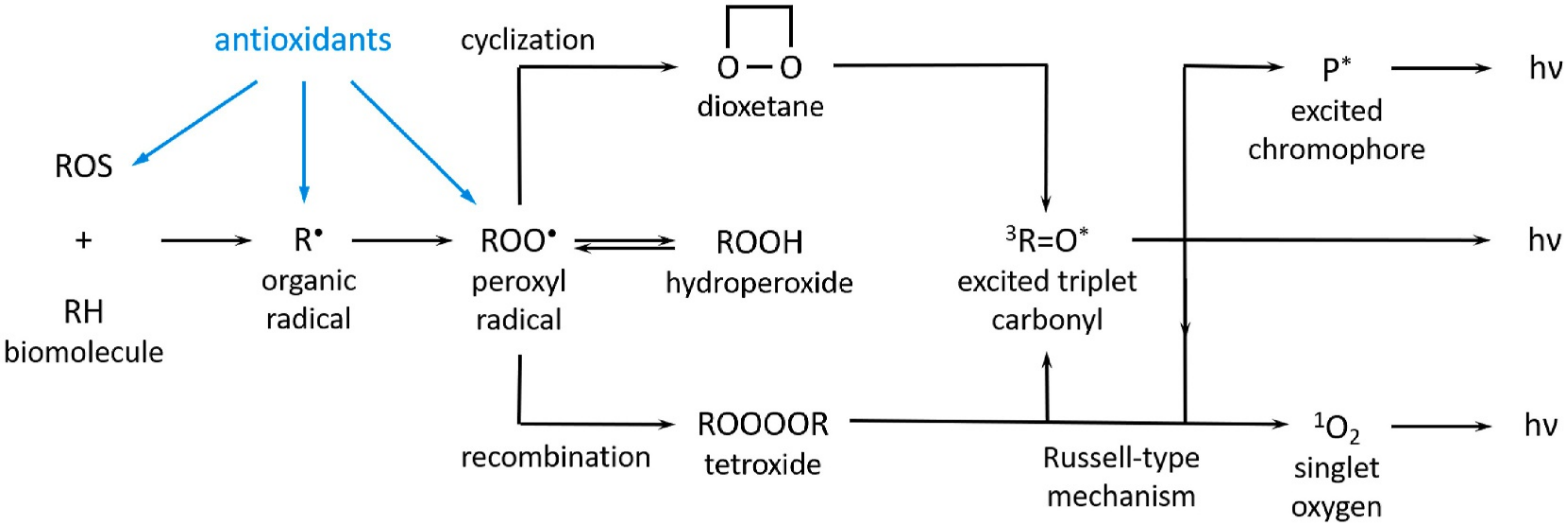
Simplified scheme of the radical chemistry that generates ultra‐weak photon emission. A biomolecule (RH) is oxidised by reactive oxygen species to an organic radical (R•), which adds oxygen to form a peroxyl radical (ROO•) in equilibrium with the corresponding hydroperoxide (ROOH). Peroxyl radicals either cyclise to an unstable dioxetane or recombine to a tetroxide (ROOOOR); fragmentation of these high‐energy intermediates (Russell‐type mechanism) generates electronically excited products excited chromophores (p*), triplet carbonyls (^3^R=O*) and singlet oxygen (^1^O_2_) that relax radiatively to emit photons (hν). Antioxidants suppress emission by intercepting the radical chain. Reprinted from P. Vahalová and M. Cifra, ‘Biological autoluminescence as a perturbance‐free method for monitoring oxidation in biosystems,’ *Progress in Biophysics and Molecular Biology*, vol. 177, pp. 80–108, Copyright (2023), with permission from Elsevier [30].

At the level of core metabolism, glycolysis and the tricarboxylic acid cycle, oxidise carbohydrates, fatty acids and amino acids to CO_2_, producing NADH and FADH_2_ as the main reduced electron carriers. In eukaryotes, these reducing equivalents feed the mitochondrial ETC, where electrons are transferred from NADH and FADH_2_ to molecular oxygen via a series of redox‐active Complexes (I‐IV) and mobile carriers (ubiquinone/ubiquinol and cytochrome *c*) [42, 43]. The exergonic nature of these redox steps is harnessed to pump protons across the inner mitochondrial membrane, establishing a proton motive force (Δp) that drives ATP synthesis via the F_1_F_0_‐ATP synthase.

Even in well‐coupled mitochondria, a small fraction of the electron flux leaks prematurely to oxygen, generating superoxide ($O_{2}^{-}$) at distinct sites within the ETC (notably Complexes I and III) and in ancillary flavoprotein dehydrogenases [22, 41]. Superoxide is rapidly dismutated to hydrogen peroxide (H_2_O_2_) either spontaneously or via superoxide dismutases; in the presence of transition metals, H_2_O_2_ can undergo Fenton and Haber–Weiss chemistry to yield hydroxyl radicals (⋅OH). Additional ROS and reactive nitrogen species (RNS) arise from NADPH oxidases, xanthine oxidoreductase, uncoupled nitric oxide synthase, peroxisomal oxidases and other oxidoreductases [2] (Figure 3). The overall result is a complex, compartmentalised ROS network, whose fluxes are usually tightly controlled by antioxidant systems but can be strongly amplified under stress.

**FIGURE 3:**
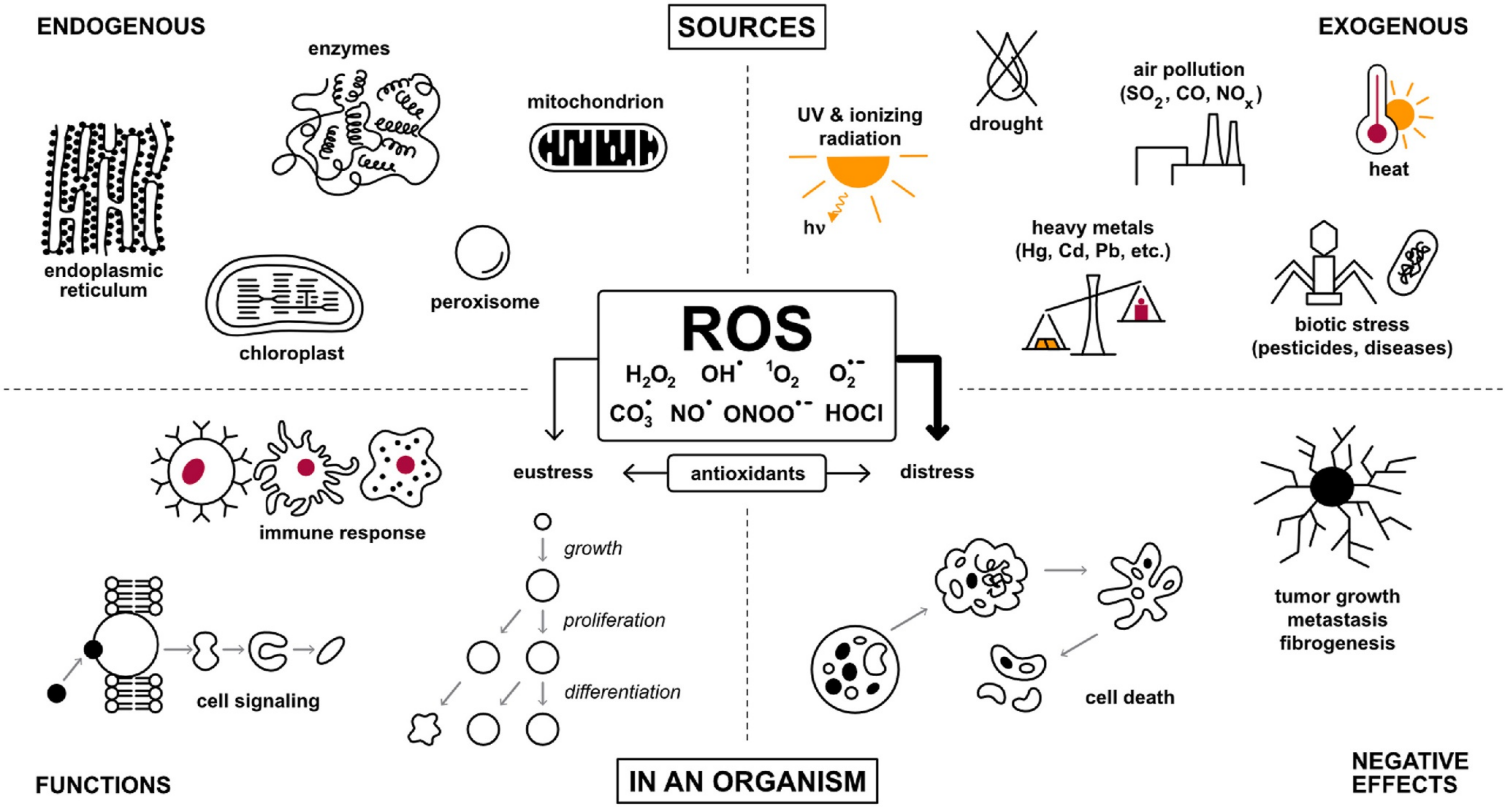
Endogenous and exogenous sources of ROS and associated effects, both functional and negative, within an organism. Reprinted from P. Vahalová and M. Cifra, ‘Biological autoluminescence as a perturbance‐free method for monitoring oxidation in biosystems,’ *Progress in Biophysics and Molecular Biology*, vol. 177, pp. 80–108, Copyright (2023), with permission from Elsevier [30].

These primary ROS in turn initiate secondary oxidative processes that are central to UPE. Polyunsaturated fatty acids in membranes are particularly susceptible to peroxidation. Initiation (e.g., by ⋅OH) produces lipid radicals (L⋅), which react with O_2_ to form lipid peroxyl radicals (LOO⋅). Propagation steps generate lipid hydroperoxides (LOOH) and new radicals; termination steps involve radical‐radical recombination and the breakdown of unstable peroxides [2, 3]. Several of these termination reactions proceed via high‐energy intermediates such as dioxetanes, dioxetanones and tetroxides, whose decomposition yields electronically excited carbonyls in triplet states (^3^C*) (Figure 4).

**FIGURE 4:**
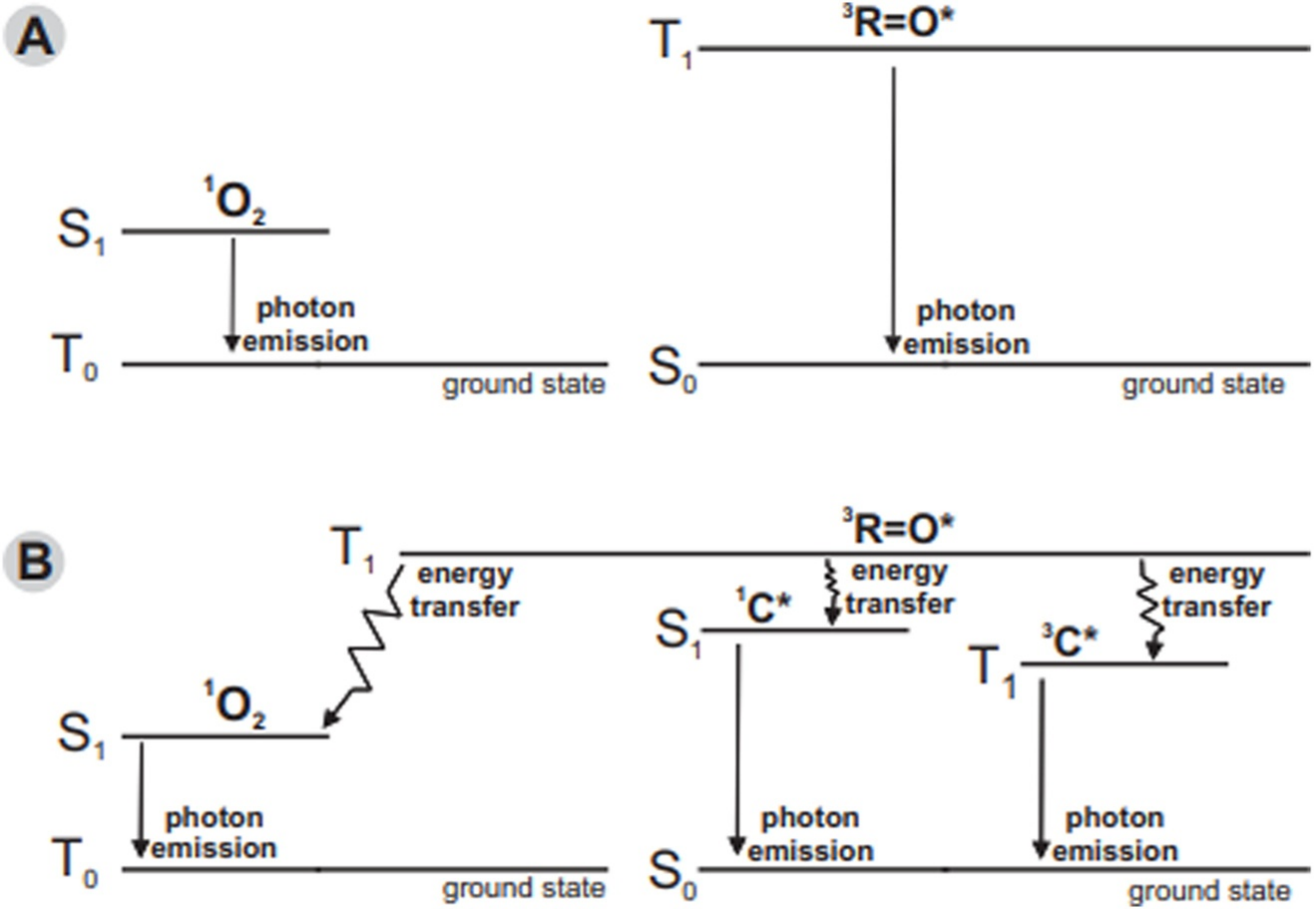
During oxidative metabolism and stress in cells, electronically excited species can form in singlet or triplet states. Reprinted from M. Cifra and P. Pospíšil, ‘Ultra‐weak photon emission from biological samples: Definition, mechanisms, properties, detection and applications,’ *Journal of Photochemistry and Photobiology B: Biology*, vol. 139, pp. 2–10, Copyright (2014), with permission from Elsevier [4].

Radiative relaxation of triplet carbonyls (^3^C* → C + hν) emits in the near‐UVA to blue, ∼380–450 nm, peaking near 430 nm [4, 44]. Emission at longer wavelengths does not come from the carbonyl itself but from energy transfer to secondary acceptors: to flavins, which fluoresce near 530 nm [45]; to porphyrins and chlorophylls, which emit in the red, ∼630–740 nm [4]; or to ground‐state O_2_, forming singlet oxygen [44].

Singlet oxygen (^1^O_2_) is another key excited species generated in oxidative environments. It can be formed via energy transfer from excited carbonyls to ground‐state O_2_, via reactions of H_2_O_2_ with hypochlorite (in neutrophils), or via photochemical processes in pigment‐rich tissues [3, 46]. ^1^O_2_ relaxes radiatively via a weak monomolecular band near 1270 nm and dimolecular (‘dimol’) bands near 634 and 703 nm when two ^1^O_2_ molecules interact [46, 47, 48]. These characteristic wavelengths recur in many reported UPE spectra and provide useful fingerprints of ROS driven chemiexcitation (Table 2).

**TABLE 2:** Representative free‐energy drops in the respiratory chain and corresponding idealised single‐photon energies, compared with characteristic ROS‐linked emission bands. Photon energies are per electron, the operative quantity for a single radiative event. Values are upper bounds: in vivo, most free energy is partitioned into Δp and heat. The formal two‐electron NADH → O_2_ sum, 2.28 eV (544 nm), is inaccessible to any one‐electron transfer and is not realised by any transition in the chain.

Transition/process	$\Delta E^{{}^{\circ}\prime }$	$\Delta G^{{}^{\circ}\prime }$	$E_{\gamma }$ (per e^−^)	Observed band
	(V)	(kJ mol^−1^, per 2e^−^)	(eV)/λ (nm)	λ (nm)
NADH → O_2_	1.14	220	1.14/∼1090	—
FADH_2_ → O_2_	0.79	152	0.79/∼1570	—
cyt c → O_2_	0.57	110	0.57/∼2180	—
cyt c → O_2_ (after 2H^+^ pumped)	—	70–80	0.36–0.41/∼3000–3400	—
^1^O_2_ dimol bands	—	—	—	∼634, ∼703
^1^O_2_ monomol band	—	—	—	∼1270
Triplet carbonyl chemiluminescence	—	—	—	∼380–450 $\left(\lambda_{\max}\approx 430\right)$

Proteins and nucleic acids are also targets of oxidative modification. Oxidation of amino acid side‐chains (e.g., tryptophan, tyrosine, cysteine, methionine) and backbone carbonyl formation can create new chromophores that either fluoresce weakly or participate in chemiluminescent reactions [2, 3]. Cross‐linking and aggregation alter local environments and may facilitate energy transfer processes. In DNA, oxidative lesions such as 8‐oxo‐7,8‐dihydroguanine can absorb and transfer energy, potentially contributing to UPE in the UV and visible regions, although their quantitative impact in vivo is uncertain [3].

Conceptually, the fundamental pathway to UPE under aerobic conditions can be summarised as:

$$\text{substrate}\;\text{oxidation}\to {\text{NADH/FADH}}_{2}\to \text{ETC}\to$$

ROS→lipid/proteinoxidation→

excitedcarbonyls,O21,pigments∗→hν

What differs between spontaneous and stimulated regimes is their fluxes, spatial organisation and the availability of high‐energy intermediates. In the next Section (2.2), we focus on the spontaneous regime, where the ETC is well coupled and ROS fluxes are low; Section 2.3 then considers how strong stressors amplify these same pathways to yield readily detectable visible UPE.

### 2.2 Spontaneous UPE From Well‐Coupled Oxidative Phosphorylation

In respiring eukaryotic cells under near homeostatic conditions, a significant source of UPE is expected to be mitochondrial oxidative phosphorylation and its associated, tightly regulated ROS turnover; this is the mitochondrial contribution that the energy budget analysis of Section 3 bounds. In this spontaneous regime, mitochondria operate close to their physiological set point: electron transport is well coupled to ATP synthesis, proton leak is modest, antioxidant systems efficiently buffer ROS, and damage repair pathways keep lipid and protein oxidation at low steady state levels [2, 22]. UPE is therefore a low flux by‐product of oxidative metabolism in a cell at rest.

Even under well‐coupled (‘physiological’) oxidative phosphorylation, electron leak and ROS production occur at a constant low flux regardless of physiological demand and participate in redox signalling; ‘non‐ideal’ conditions primarily reflect overproduction and/or mislocalisation that overwhelms buffering.

The relevant pathway as outlined in Section 2.1:

ETCelectronflux→smallROSleak→

low‐levellipid/proteinoxidation→

rarechemiexcitedintermediates→hν

Because ROS production is limited and most oxidation events are intercepted or repaired before propagating, there is only a small fraction of the respiratory free energy that enters radiative channels. Quantitative energy budget arguments, outlined in Section 3, show that for mitochondria, respiratory rates and coupling efficiencies, the corresponding photon yields must be extremely low many orders of magnitude below typical fluorescence or bioluminescence signals and often close to or below the sensitivity limits of standard detectors for practical sample geometries and integration times.

The spectral characteristics of spontaneous UPE are likewise constrained by mitochondrial energetics and ROS chemistry. Direct radiative loss from the respiratory chain is excluded from the visible and from the NIR below ∼2000 nm (Section 3.2): every individual redox step lies beyond ∼2000 nm, and the terminal residual after proton‐pumping beyond ∼3000 nm. Once delayed luminescence and instrumental artefacts are excluded, what is detected from a resting cell is therefore basal chemiexcitation driven by physiological ROS leak, emitted through the same species that dominate under stress: triplet carbonyls near 430 nm, flavins and pigments excited by energy transfer, and singlet oxygen at 634, 703 and 1270 nm [4, 44, 46]. The two regimes differ chiefly in flux. One spectral consequence follows from the kinetics: dimol ^1^O_2_ emission is second order in [^1^O_2_], so the 634 and 703 nm bands gain weight relative to first‐order emitters as ROS flux rises [46, 47, 48].

Experimentally, this spontaneous regime is challenging to probe. In healthy tissues, spontaneous UPE is heavily attenuated by scattering and absorption, further reducing the already low photon flux that reaches a detector. Imaging detectors (CCD, EMCCD, Scientific Complementary Metal‐Oxide‐Semiconductor (sCMOS)) accumulate dark current and read noise over the long integration times needed to collect sufficient signal, whereas single channel photon counters (PMTs, single photon avalanche diode (SPADs)) must contend with dark counts and stray light backgrounds that can match or exceed the expected signal [4, 19, 49]. This suggests many published ‘spontaneous UPE’ studies operate in a regime where stimulatory factors (e.g., sample handling, ambient light, subtle stressors) make nonspontaneous contributions, even when no explicit stress is applied.

Within this review, we therefore adopt the following working definition: spontaneous UPE from well‐coupled oxidative phosphorylation denotes the ultra‐weak photon emission that arises from basal chemiexcitation when mitochondria are efficiently converting substrate free energy into Δp and ATP, ROS are maintained at low steady‐state levels and oxidative damage is minimal. This is the regime that the energy budget model of Section 3 quantifies. It provides the reference baseline against which we interpret stronger UPE signals: large increases in intensity, especially emission detectable on experimentally convenient timescales, are most naturally read as signatures of the stimulated, high‐ROS regimes discussed in Sections 2.3 and 2.4.

It is useful to separate (i) energetic constraints on any direct radiative loss tied closely to mitochondrial redox steps from (ii) secondary oxidation chemistry that generates electronically excited intermediates. Under a single‐step energetic picture, any direct emission would be confined to wavelengths beyond ∼2000 nm (Section 3.2) and would represent only a small fraction of the total dissipated energy. By contrast, most reported visible UPE is more consistently explained by secondary chemistry (e.g., triplet excited carbonyls, singlet oxygen pathways including dimol bands, excited pigments, and DL following illumination). Throughout the review, we treat measured UPE as a reporter of oxidative chemistry; any direct energetic component would lie in the mid‐infrared, beneath the sample's own thermal emission [3, 4, 12, 22, 30].

### 2.3 Stimulated and Pathological UPE: Stressors, Lipid Peroxidation, Delayed Luminescence

The majority of experimentally accessible UPE signals arise when biological systems are driven far from homeostasis by physical or chemical stressors. In the stimulated regime, photon emission is dominated by large, transient bursts of ROS and RNS, membrane damage, lipid peroxidation and, in some cases, delayed relaxation of photochemically primed states. Strong, visible‐range UPE is therefore most naturally interpreted as a hallmark of high‐stress or pathological chemistry, rather than a direct readout of spontaneous mitochondrial respiration.

A range of stimuli have been shown to evoke pronounced UPE. UV and blue visible irradiation of cells, tissues or skin consistently produces orders of magnitude increases in photon emission that decay over seconds to hours after the end of illumination [23, 50, 51, 52]. Photons absorbed by endogenous chromophores (porphyrins, flavins, melanin, advanced glycation and lipoxidation end products) generate excited states and radical pairs that initiate ROS cascades. Superoxide, hydrogen peroxide and hydroxyl radicals then attack membrane lipids, proteins and nucleic acids, driving lipid peroxidation chain reactions and protein oxidation. These pathways populate high energy intermediates such as dioxetanes, dioxetanones, tetroxides and triplet carbonyls, as well as singlet oxygen (^1^O_2_), all of which can relax radiatively with emission spanning the visible and NIR [3, 44].

Comparable chemistry occurs in ischemia‐reperfusion and mechanical injury models. Sudden reoxygenation of hypoxic tissues, or physical disruption of membranes, leads to intense ROS production at mitochondria and plasma membranes, culminating in lipid breakdown, protein cross‐linking and cell death. UPE from ischemic hearts, brain slices and mechanically injured tissues shows large, transient increases with spectra consistent with lipid peroxidation and ^1^O_2_ emission [5]. In vivo exercise studies provide another example: strenuous physical activity, triggers oxidative bursts in muscle and several groups have reported elevated UPE from skin overlying active muscle groups post exercise [53]. In each case, the key commonality is a substantial departure from spontaneous ROS homeostasis.

Plant systems display analogous behaviour. Senescing leaves, pathogen infected tissues and plants exposed to drought, extreme temperatures or mechanical wounding exhibit strong increases in UPE relative to healthy controls [54, 55, 56]. Emission intensities can rise by several orders of magnitude as chlorophyll degrades, membranes disintegrate and programmed cell death progresses. Spectrally, plant UPE often shows visible bands associated with chlorophyll‐derived intermediates and ROS chemiluminescence, again pointing to oxidative stress as the primary driver rather than steady‐state photosynthesis or respiration [57].

DL is the nonstationary component that decays after prior photoexcitation; spontaneous UPE is the stationary floor remaining after dark adaptation. DL represents a specific, and widely studied, manifestation of this stimulated regime. Following a period of photic excitation, many biological samples including photosynthetic tissues, mammalian cells and isolated organelles emit photons for milliseconds to minutes, with decay curves that are typically hyperbolic or stretched exponential. In photosynthetic organisms, DL is closely linked to recombination processes and metastable charge‐separated states in the photosystems and associated pigment‐protein complexes. In nonphotosynthetic systems, DL has been attributed to recombination within the respiratory chain, persistent radical pairs and long‐lived excited states in chromophoric macromolecules. DL requires prior energetic priming and reflects the gradual relaxation of a perturbed system; it does not occur in the absence of an initial stimulus.

Together, these observations support a simple but important message: whenever strong, readily detectable visible UPE is observed from cells, tissues or organisms, the system is almost always in a stimulated regime characterised by large ROS bursts, lipid and protein oxidation, membrane damage, cell death or prior photic priming. Under such conditions, the measured emission is dominated by ROS linked chemiexcitation and DL, not the spontaneous glow of well‐coupled oxidative phosphorylation described in Section 2.2. This distinction is central for interpreting “biophoton” data throughout the rest of this review. Photoinduced delayed luminescence decays over seconds to minutes after illumination and can be mistaken for spontaneous emission [4]. Studies claiming spontaneous emission should therefore report dark‐adaptation duration and demonstrate that emission has reached a plateau; residual monotonic decay indicates DL contamination rather than a spontaneous signal.

Oxygen‐delivery perturbations: occlusion‐reperfusion as a canonical stressor control is a useful, reagent free physiological perturbation is transient ischaemia induced by arterial occlusion, which directly modulates oxygen delivery and tissue redox state. In a human protocol combining PMT‐based UPE detection with NIR spectroscopy, Scholkmann et al. (2013) [58] reported that UPE decreases significantly during arterial occlusion (but not venous occlusion) and rises after cuff release during reperfusion, with UPE showing a significant (nonlinear) association with haemodynamic/oxygenation surrogates (oxy‐ and deoxy‐haemoglobin and their difference). These findings make oxygen delivery perturbation a clear ‘stressor’ exemplar within the broader oxidative process framework for UPE. The occlusion–reperfusion paradigm is valuable as a canonical positive control for low photon instrumentation, because it provides a time‐locked, reversible modulation of physiological state without introducing exogenous luminophores or injection artefacts. In practice, the same standardised cuff protocol can be used to benchmark detector modalities (e.g., PMT, SPAD, EMCCD) on identical tissue with matched shielding and analysis, reporting effect sizes and time constants rather than relying only on absolute count changes. Although UPE changes may be entangled with haemodynamic and optical property changes during occlusion, the within‐subject reversibility and the reported directionality (decrease during occlusion; increase on release) make it a pragmatic end‐to‐end validation of optics, shielding, detector stability and drift/control‐channel analysis [58]. Reperfusion is expected to introduce a distinct redox transient (often described as an oxidative burst driven by mitochondrial ROS mechanisms), but the direction and magnitude of any UPE transient can depend on protocol details and tissue state [59, 60]. This makes reperfusion a natural follow on condition for time resolved BAL/UPE controls, especially when paired with simultaneous oxygenation/haemodynamic readouts.

#### 2.3.1 Membrane and Metabolic Perturbations → ROS → UPE

Consistent with this, a range of membrane and metabolic perturbations modulate UPE through the same ROS chemistry. Heat shock elevates UPE via oxidative stress and lipid peroxidation [61]. Exogenous oxidants such as H_2_O_2_ reliably increase UPE in cells and tissues [3]. In yeast, DL covaries with mitochondrial morphology, oxygen consumption rate and membrane potential, coupling photonic afterglow to bioenergetic state [62]. ETC inhibitors (e.g., antimycin A, rotenone) reshape ROS generation and membrane potential [63], implying predictable (but still to be directly mapped) UPE shifts via the same ROS→UPE chemistry [3]. Together these results support a working model that interventions altering inner mitochondrial membrane integrity, proton motive force (Δp), or redox buffering will, via ROS, modulate UPE.

### 2.4 Modulation by Antioxidants and Bystander Effects

The strong oxidative component of stimulated UPE is underscored by its sensitivity to antioxidant status. Broadly, exogenous and endogenous antioxidants which reduce ROS generation, scavenge radicals or terminate lipid peroxidation chains tend to suppress UPE intensity and alter its temporal and spectral characteristics [2, 15, 41]. Conversely, depletion or inhibition of key antioxidant systems often enhances emission.

Vitamins C and E, glutathione (GSH) and its precursors, carotenoids and polyphenolic compounds are among the most widely studied modulators. In UV exposed skin, topical application of vitamin C/E formulations or antioxidant rich plant extracts reduces UV induced UPE amplitude and accelerates its decay, consistent with quenching of radicals and excited intermediates [52, 64, 65]. In cell cultures and isolated organelles, boosting GSH levels or overexpressing antioxidant enzymes (superoxide dismutases, catalase, peroxiredoxins) similarly attenuates UPE responses to oxidant challenges, whereas pharmacological or genetic depletion of these defences often leads to enhanced or prolonged emission [2]. In plant and seed systems, antioxidant‐rich varieties or treatments generally show lower UPE under equivalent stress, reflecting more effective containment of lipid peroxidation and ROS cascades [18, 66, 67].

The relationship between antioxidants and UPE is not always a simple monotonic suppression. Some compounds possess their own excited‐state dynamics and may act as physical quenchers or alternative emitters; others can become pro‐oxidant at high concentrations or in the presence of transition metals, thereby increasing ROS production and, paradoxically, UPE [2]. Furthermore, spectral filtering and re‐emission effects can complicate interpretation in the visible range: strong absorption, fluorescent antioxidants may cause attenuation or reshape observed spectra without necessarily changing the underlying chemiexcitation rates. Robust conclusions therefore require careful control experiments and, ideally, independent assessment of ROS and oxidative damage markers alongside UPE.

Beyond local modulation, stimulated UPE also participates in broader patterns of bystander effects and stress propagation. In cell culture, photons emitted by irradiated cells have been reported to induce bystander responses, including exosome release, in unirradiated cells exposed only to that emission [68].

Mechanistically, these nonlocal responses are most plausibly explained by diffusing chemical messengers (ROS/RNS, cytokines, danger associated molecular patterns), calcium waves, gap‐junction coupling and electrophysiological signals, rather than by photons themselves acting as primary messengers [69, 70]. Photons may contribute as one of several overlapping pathways, but the dominant drivers of bystander stress are chemical and electrical. In this context, UPE serves as an optical correlate of a spreading oxidative wave: an easily measurable reporter of where and when stress has propagated, without necessarily mediating that propagation.

Viewed through the two‐regime framework, antioxidant sensitivity and bystander phenomena are not peripheral curiosities but integral aspects of the stimulated UPE regime. They reinforce the interpretation of UPE as a responsive marker of oxidative network dynamics highly sensitive to changes in redox buffering and capable of revealing spatial patterns of stress propagation while simultaneously underlining its dependence on conditions far removed from spontaneous, well‐coupled oxidative phosphorylation.

### 2.5 Summary: UPE as a By‐Product

The literature reviewed in Sections 2.2–2.4 motivates a two‐regime view of biological UPE. In the spontaneous regime, well‐coupled oxidative phosphorylation and routine ROS turnover produce extremely weak emission, through basal chemiexcitation, that is tightly constrained by the mitochondrial energy budget and likely to lie at or below the sensitivity limits of standard detectors for realistic sample geometries and integration times. In the stimulated regime, strong perturbations (UV/visible irradiation, chemical oxidants, ischaemia‐reperfusion, mechanical damage, senescence and cell death, intense exercise and other stressors) drive large ROS bursts, lipid peroxidation cascades, and long lived excited intermediates, yielding orders of magnitude higher photon fluxes, often with prominent visible components that are readily detectable and strongly modulated by antioxidant status and stress propagation.

Beyond the canonical ROS leak from Complexes I and III, mitochondria can generate $O_{2}^{\cdot -}/H_{2}O_{2}$ at several other redox enzymes most notably the oxoacid dehydrogenase complexes (pyruvate dehydrogenase and α‐ketoglutarate dehydrogenase) and additional flavin/ubiquinone linked dehydrogenase; so, the significant ROS source depends strongly on substrates and bioenergetic state [22, 71, 72].

Within this framework, UPE is best regarded as a by‐product of oxidative metabolism that becomes optically salient primarily when biological systems are pushed away from homeostasis. This has direct consequences for interpreting biophoton data: robust visible UPE is far more plausibly read as a signature of high ROS, high damage chemistry than as the direct output of routine mitochondrial respiration, and any proposed physiological roles for spontaneous UPE must operate under the severe photon budget and spectral constraints implied by well coupled oxidative phosphorylation. The next section translates these qualitative distinctions into quantitative limits, using the mitochondrial energy budget to constrain feasible photon yields and spectral distributions under spontaneous conditions (Section 3).

## 3 Quantitative Constraints on Mitochondrial UPE

Section 2 outlined the oxidative pathways and excited intermediates that give rise to UPE. Several proposals attribute functional roles to photons emitted by mitochondria or the respiratory chain: coherent biophoton fields [6, 7], mitochondrial biophotons guided along microtubules to influence membrane activity and carry neural information [73, 74], and optical communication channels between neurons [8, 75]. Such proposals require that respiration deliver visible photons at yields and energies sufficient to signal. Section 2 established that the measured signal is dominated by secondary ROS chemiexcitation. Section 3 asks: how much energy is available for a single, direct radiative transition along the respiratory chain and at what wavelength? The energy budget bounds what direct ETC emission is possible; it does not itself produce the observed signal, which arises from the chemistry of Section 2.

Taking the mitochondrial ETC of *S. cerevisiae* as a representative eukaryotic system, we use standard redox potentials and proton‐pumping stoichiometries to estimate the free‐energy drop at each step and the fraction captured in ATP synthesis. Residual energy is dissipated as heat or, rarely, as a photon if an appropriate excited intermediate is formed. The analysis sets upper bounds on the intensity and wavelength of direct ETC emission from well coupled respiration, and distinguishes it from ROS driven chemiexcitation.

### 3.1 Energy Budget of the Mitochondrial ETC (Yeast as a Model)

Here we ask whether any single radiative transition along the ETC can produce a photon in the visible range. A visible photon requires an energy of approximately 1.8 eV (≈690 nm, red) to 3.1 eV (≈400 nm, violet). We therefore compare this threshold against the free energy available at each individual redox transition of the respiratory chain.

The energy budget developed here is based on the respiratory chain of *S. cerevisiae*, in a nonfermentative state (definition of the spontaneous regime, Section 1.3). Glucose‐grown *S. cerevisiae* undergo strong fermentative metabolism through the Crabtree effect, during which mitochondrial respiration is suppressed; such cultures are not representative of the resting respiratory state and are not appropriate for the spontaneous baseline considered here. *S. cerevisiae* lacks a canonical Complex I; NADH is oxidised instead by nonproton‐pumping alternative dehydrogenases (internal Ndi1, external Nde1/Nde2), whereas Complexes II–IV are retained [74, 75, 76, 77, 78, 79, 80]. This changes the total H^+^ pumped per 2e^−^ and the P/O ratio, but neither enters the bound derived below. The midpoint potentials of NAD^+^/NADH, fumarate/succinate, cytochrome c and O_2_/H_2_O are the same in yeast and mammals, and Complex IV pumps 2 H^+^ per 2e^−^ in both; the constraint on photon energy is therefore independent of organism.

Oxidative phosphorylation converts the free energy of substrate oxidation into a proton motive force (Δp) and ATP. UPE drawn from this process must therefore compete with the dominant, highly efficient channels of proton‐pumping and ATP synthesis. To set quantitative limits on spontaneous mitochondrial UPE, it is useful to articulate the energy budget of the respiratory chain in a concrete model system such as *S. cerevisiae*.

Aerobic glucose oxidation in *S. cerevisiae* yields reducing equivalents, distributed between NADH and FADH_2_. Pyruvate dehydrogenase and the tricarboxylic acid cycle generate matrix NADH and FADH_2_, whereas glycolytic NADH is re‐oxidised via external dehydrogenases that feed electrons directly into the ubiquinone pool [76, 77]. Unlike mammalian mitochondria, yeast lack a proton‐pumping Complex I; instead, internal (Ndi1p) and external (Nde1/2p) NADH dehydrogenases are nonpumping, and proton translocation occurs only at Complexes III and IV. For each pair of electrons passed from reduced ubiquinol (QH_2_) to oxygen via III and IV, approximately 6H^+^ are pumped (4 at Complex III and 2 at Complex IV), compared to ∼10H^+^ per 2e^−^ in mammalian complex I‐III‐IV chains [43].

The standard free energy change for NADH oxidation by oxygen,

$$NADH+\frac{1}{2}\;O_{2}\to NAD^{+}+H_{2}O,$$

is on the order of $\Delta G^{◦\prime }\approx -220kJ\;mol^{-1}$ for the 2e^−^ pair [42]. This is sufficient, in a mammalian‐type chain, to pump roughly 10 H^+^ across the inner membrane and support a P/O ratio of ∼2.5 ATP per NADH. FADH_2_ enters at nonpumping Complex II, passes through Complexes III and IV, and releases ≈−152 kJ mol^−1^ per 2e^−^, driving ∼6H^+^ and yielding a P/O ratio of ∼1.5 ATP [81]. In yeast, the absence of Complex I lowers the total number of protons pumped per substrate oxidised and thus the ATP yield, but the overall topology of the energy budget remains similar.

At the level of individual steps, the terminal cytochrome *c* oxidase reaction

$$4\;{cyt\;c}_{\left(red\right)}+O_{2}+8\;H_{\left(matrix\right)}^{+}\to 4\;{cyt\;c}_{\left(ox\right)}+2\;H_{2}O+4\;H_{\left(IMS\right)}^{+}$$

corresponds to a redox drop of $\Delta G^{◦\prime }\approx -110kJ\;mol^{-1}$ per 2 e^−^, based on the difference between the midpoint potentials of cytochrome *c* ($E^{◦\prime }\approx +0.25$ V) and O_2_/H_2_O ($E^{◦\prime }\approx +0.82$ V) [82]. At a typical proton motive force of Δp≈170‐200 mV, pumping 2H^+^ at Complex IV consumes roughly 30–40 kJ mol^−1^, with the remainder dissipated as heat and, only in exceptionally rare events, as radiation.

Expressed in energetic terms, each NADH delivers ∼220 kJ mol^−1^, of which ∼50–60 kJ mol^−1^ are stored in ATP (2‐3 ATP at ∼20 kJ mol^−1^ per ATP in vivo), and the rest is dissipated [42, 83]. In tightly coupled mitochondria, most of this dissipation occurs as distributed heat at proton‐pumping sites and in leak pathways, not as a single radiative transition. Thus, the fraction of the respiratory energy budget that is both available at the terminal step and capable of emerging as photons is extremely small. In yeast, with lower proton‐pumping stoichiometry and altered dehydrogenase architecture, the same conclusion holds: spontaneous mitochondrial UPE must draw on a tiny residue of the overall oxidative budget.

This energy budget describes the spontaneous regime defined in Section 2.2: well‐coupled respiration in which ROS production is low and redox free energy is overwhelmingly channelled into Δp and ATP, leaving minimal radiative loss.

### 3.2 Expected Photon Yields and Spectral Distributions

Relating this energy budget to potential photon emission requires converting representative free‐energy drops into photon energies and wavelengths. For a redox step transferring n electrons across a potential difference $\Delta E^{◦\prime }$, the energy available per electron is

$$E_{\gamma }=\frac{\left|\Delta G^{◦\prime }\right|}{nN_{A}}$$

where $N_{A}$ is Avogadro's number and e the elementary charge, so that $E_{\gamma }$ in electronvolts equals $\Delta E^{◦\prime }$ in volts. Using $E_{\gamma }=hc/\lambda$ with hc≈1240eVnm,

$$E_{\text{eV}}=\frac{1240}{\lambda_{\text{nm}}}.$$

Redox spans are conventionally tabulated per two electrons, the stoichiometry of NADH and succinate oxidation and of O_2_ reduction to water. Radiative decay is a single‐quantum event, and inter‐cofactor transfer proceeds by one‐electron steps: flavins, iron–sulfur clusters and haems each carry one electron, and ubiquinone is reduced in two sequential transfers. No photon can carry the two‐electron sum. Per‐electron values are used throughout; the two‐electron NADH → O_2_ total is given in the caption to Table 2 as a formal sum only.

Converting each free‐energy drop to a photon energy (Table 2), the whole chain NADH → O_2_ span, $\Delta E^{◦\prime }=1.14$ V, gives $E_{\gamma }=1.14$ eV per electron (λ≈1090 nm), a formal ceiling that no single transfer realises. The largest individual step, cytochrome c → O_2_, spans 0.57 V, or 0.57 eV per electron (λ≈2180 nm).

Accounting for the 2 H^+^ pumped at Complex IV reduces the free energy available for any direct radiative event further. Subtracting the ∼30–40 kJ mol^−1^ used to move protons against Δp leaves ∼70–80 kJ mol^−1^ per 2e^−^, or 0.36–0.41 eV per electron, corresponding to λ≈3000–3400 nm. Under realistic coupling, any photonic leakage from the terminal ETC step is mid‐IR.

Table 2 summarises these reference values and juxtaposes them with known chemiluminescent signatures of reactive oxygen species. Two conclusions follow.

1. No ETC transition can emit in the visible. The most energetic single transition available is cytochrome $c\to O_{2}$ at 0.57 eV per electron (∼2180 nm); the whole chain NADH to oxygen span of 1.14 eV per electron (∼1090 nm) is a formal ceiling that no single transfer realises. After proton pumping at Complex IV the terminal residual is 0.36–0.41 eV, beyond ∼3000 nm. This holds even assuming the entire one electron drop appears as a photon rather than being dissipated.
2. Green or blue photons (λ≤ 550 nm, E≥ 2.25 eV) would require the entire two electron NADH $\to O_{2}$ free energy to be delivered to a single radiative event. This is not merely incompatible with well‐coupled oxidative phosphorylation; it is inaccessible to any one electron transfer, and so cannot occur by a direct ETC route under any coupling condition.

By contrast, ROS linked chemiluminescent pathways generate higher energy photons through specific high energy intermediates. Triplet carbonyls formed during lipid peroxidation, dioxetane and tetroxide fragmentation, and singlet oxygen (^1^O_2_) produced by energy transfer from excited pigments or radicals all give rise to characteristic visible and NIR bands [84, 85]. The dimol ^1^O_2_ bands at ∼634 and ∼703 nm and the monomol band near 1270 nm are well established spectral signatures of oxidative chemiexcitation and match many reported features of UPE spectra under stress [46, 47, 48].

Given that only a small fraction of respiratory turnovers can give rise to photons, spontaneous photon yields tied to ETC operation must be very low. Visible emission requires excited intermediates that lie outside the proton‐pumping chemistry of the ETC: triplet carbonyls, singlet oxygen, and pigments excited by energy transfer from these species. Their population scales with ROS flux and rises sharply during ROS bursts, lipid peroxidation cascades and other high‐stress processes, particularly the intermediates produced during ROS bursts, lipid peroxidation cascades and other high stress processes.

The key result follows directly: no individual ETC redox transition spans sufficient free energy to produce a visible photon. The largest individual step, cytochrome c → O_2_, corresponds to ∼2180 nm, and even the whole‐chain NADH‐to‐oxygen span, 1.14 eV per electron (∼1090 nm), lies beyond the ∼690 nm red limit of Section 3.1. Visible photons attributed to mitochondria cannot originate in a direct ETC redox transition and must arise from the secondary ROS chemistry of Section 2, which reports oxidative chemistry rather than a respiratory signalling channel.

### 3.3 Implications for Interpreting Reported UPE Intensities and ‘Biophoton’ Claims

The thermodynamic constraints of Section 3.2 impact the interpretation of UPE data. Under spontaneous, well‐coupled oxidative phosphorylation, only a very small fraction of the respiratory free energy can plausibly appear as photons, and none of it at visible or NIR wavelengths by a direct route. The largest single redox step, at cytochrome c oxidase, corresponds to ∼2180 nm; after proton‐pumping at Complex IV, any direct ETC‐linked emission lies beyond ∼3000 nm.

Direct ETC emission is therefore both extremely weak and spectrally inaccessible. The band in which it is predicted is dominated by the sample's own thermal emission: at 310 K a sample radiates of order $4\times 10^{11}$ photons s^−1^ cm^−2^ per 100 nm band at 2180 nm, and $10^{13}$–$10^{14}$ at 3000–3400 nm [86, 87], against reported UPE intensities of $10^{-1}$–$10^{2}$ photons s^−1^ cm^−2^ [4, 88, 89, 90]. Unlike instrumental backgrounds, this floor cannot be removed by cooling the detector, since the sample is at physiological temperature by definition (Section 4.3.1). The constraint on direct ETC emission is thus thermodynamic rather than experimental: no measurement on a sample at physiological temperature can test it.

Visible emission requires secondary chemiexcitation: the generation of triplet carbonyls and singlet oxygen through downstream ROS chemistry, in which multi‐step radical reactions populate excited states. The ETC is the source of the ROS that drive this chemistry but is not itself a direct emitter.

This is the foundation of the two‐regime framework (Section 1.3). The ETC of a cell at rest is radiatively silent in the visible: no direct redox transition can emit there. Visible UPE from a resting cell therefore reports basal chemiexcitation driven by physiological ROS leak, not respiration itself. Departures from rest (elevated mitochondrial activity, as in exercise; external oxidative challenge; or disease) raise the ROS flux driving the same chemistry, increasing emission intensity.

This view differs from the stronger ‘biophoton’ claims in the literature, in which robust UPE is reported from ostensibly healthy cells or tissues, often with pronounced blue‐green components and relatively high photon yields per unit oxygen consumption [85, 91]. Within the framework developed here, high UPE is better understood as an indicator of ROS linked chemiexcitation at elevated flux than as a readout of routine respiration. Large increases in UPE intensity and the emergence of structured visible spectra are most commonly observed under strong perturbation: UV or blue‐light irradiation, chemical oxidants, ischaemia‐reperfusion, mechanical injury, senescence and cell death or intense exercise [50, 51, 53, 58]: conditions in which UPE reflects ROS bursts and membrane breakdown, and is strongly modulated by antioxidant status and bystander stress propagation (Section 2.3). Interpreting such signals as evidence of coherent communication between healthy cells must account for the dominant role of oxidative chemistry in generating them.

In the spontaneous regime defined in Section 2.2, mitochondrial UPE is a rare by‐product of oxidative metabolism, emitted through the same chemiexcited species at much lower flux, with intensities at or below the detection limits of most conventional photon‐counting instruments for realistic sample geometries and integration times. Section 4 frames these constraints as detection limits and provides criteria for judging whether a given configuration is genuinely sensitive to spontaneous UPE or is probing stimulated, ROS‐dominated emission.

## 4 Detection Technologies and Practical Limits

Biological UPE has broad count rates that are many orders of magnitude below those of conventional fluorescence microscopy, often at or below $10^{-1}$‐$10^{1}\;photons\;s^{-1}$ [92] from a single cell or small tissue region. The instrumental challenge is therefore not simply to detect single photons, but to do so over biologically relevant timescales with acceptable signal‐to‐noise ratio (SNR) [4, 93] in the presence of dark noise, read noise and environmental backgrounds. Across detector families, the key performance metrics are: quantum efficiency (QE), dark counts/dark current, readout noise, timing resolution, dynamic range and spatial sampling, see Table 3. In this section, the most relevant detector classes for UPE are reviewed, their strengths and limitations are highlighted, and then practical recommendations are formulated for different experimental regimes.

**TABLE 3:** Current Detector technologies used within the UPE/Biophoton field.

Detector	Peak QE	Spectral range	Dark noise (typical)	Operating temp	UPE track record
PMT	20%–40% (blue‐green); falls sharply> 650 nm	∼300–650 nm (bialkali); to 850 nm (multialkali/GaAs)	10–100 cps (cooled)	Room temp; cooled to −20 to −30°C to suppress dark counts	Workhorse of the field; single‐channel, high temporal resolution used in the majority of historical UPE studies.
Cooled CCD	40%–90% (back‐illuminated, visible)	∼350–1000 nm (Si limit)	Very low (long integration); read noise dominates	−70 to −100°C	Standard for 2D UPE imaging; requires long integration (minutes–hours).
EMCCD	Up to 90% (back‐illuminated)	∼350–1000 nm (Si limit)	Sub‐electron effective read noise via electron multiplication; clock‐induced charge is the practical floor	−60 to −100°C	Established for low‐flux UPE imaging where read noise would otherwise dominate; used in human/tissue UPEimaging.
sCMOS	60%–82%	∼350–1000 nm (Si limit)	Low read noise (∼(1‐2 e^−1^rms read noise) [94]; higher pixel‐to‐pixel variation than EMCCD	−10 to −40°C	Emerging‐but‐validated; increasingly used for UPE where large arrays and speed matter.

### 4.1 Established Detector Technologies

#### 4.1.1 Photomultiplier Tubes (PMTs)

PMTs are the archetypal ultra‐low light detectors and remain a benchmark for photon counting [95, 96, 97]. A PMT combines a photosensitive cathode with a cascaded dynode chain: each incident photon that releases a photoelectron at the cathode is multiplied through secondary emission to yield a macroscopic current pulse at the anode. Modern low noise PMTs achieve dark count rates of a few to a few tens of counts per second, with single‐photon sensitivity and sub‐nanosecond timing resolution [97, 98]. These properties make PMTs particularly attractive for UPE counting at a single spatial location or within a predetermined field of view defined by an optical fibre or imaging optics.

From an UPE perspective, the advantages and limitations of PMTs for UPE are summarised in Table 4.

**TABLE 4:** Advantages and limitations of established detector technologies for UPE measurement. Quantitative specifications are summarised in Table 3.

Detector	Advantages for UPE	Limitations for UPE
PMT	Very low dark counts; after internal gain, readout noise is negligible, so SNR is Poisson‐limited [11, 97]. True photon counting with sub‐nanosecond timing, allowing continuous kinetics over seconds to hours and time‐tagging to stimuli. Mature, well characterised and readily fibre‐coupled [14].	Point or integrating detector; imaging requires scanning or multi‐anode arrays [14]. Modest QE (peak ∼20%–40%, lower in the red and NIR) [11, 95, 99]. Red and NIR sensitivity needs specialised photocathodes; high gain limits dynamic range and risks saturation. Requires high voltage, light shielding and over‐exposure protection.
CCD	Back‐illuminated devices reach QE ≈90% in the visible, with sensitivity to ≈1100 nm [100, 101]. Deep‐cooled devices (dark current $<10^{-3}$ e^−^ pixel^−1^ s^−1^) can image strongly stimulated samples [101, 102, 103, 104, 105].	Read noise of a few e^−^ rms per readout; dark signal and hot pixels accumulate over long exposures [101]. Generally impractical as a primary detector for spontaneous UPE.
EMCCD	QE ≥ 90% with electron multiplication allows spatial mapping where a PMT gives only an integrated count [19, 99, 106, 107]. Read noise negligible after EM gain. Operable in photon‐counting (short thresholded frames) or cumulative‐integration mode.	Stochastic gain adds excess noise, so devices are not strictly quantitative at ultra‐low flux, complicating absolute yields [93]. Needs deep cooling and stable gain; cosmic rays, clock‐induced charge and drift accumulate. High gain reduces full‐well capacity and dynamic range. Limited frame rate: fast transients cost resolution or field of view.
sCMOS	High frame rates and large fields of view, suited to bright, dynamic stimulated UPE. Low read noise (1‐2 e^−^ rms; sub‐electron in recent devices) gives acceptable SNR at a few photons pixel^−1^ frame^−1^; binning is typically digital, so read noise adds in quadrature [19, 94, 108, 109]. QE ∼60%–80% [19]. No multiplication noise; linear over most of its dynamic range, with per‐pixel calibration required at low signal [94, 110].	Not optimised below ∼1 photon pixel^−1^ frame^−1^, where dark current, read noise and fixed‐pattern noise dominate [19]. Higher dark current than deep‐cooled EMCCDs. Pixel and column gain variation can mask low‐level signals [19, 108, 111, 112]. Large data volumes require binning or averaging.

PMTs have repeatedly demonstrated their ability to measure ultra‐weak bioluminescence and UPE from cell suspensions, tissues and whole organisms [14, 19]. For experiments where spatial resolution is secondary to maximising photon statistics (e.g., long‐term monitoring of bulk emission or quantitative time series from a defined region), a well‐chosen PMT coupled to low background optics remains a robust first choice.

#### 4.1.2 CCD, EMCCD and sCMOS Cameras

Solid‐state imaging sensors accumulate photoelectrons in parallel across an array of pixels, trading some ultimate sensitivity for spatial information. For UPE, three families are most relevant: standard CCDs, EMCCD and sCMOS cameras [49, 100, 113, 114, 115].

#### 4.1.3 Standard CCDs

In a front illuminated CCD, incident photons pass through gate structures before reaching the photosensitive region, leading to moderate QE and additional scattering. In back thinned, back‐illuminated CCDs, the silicon substrate is thinned and illuminated from the rear, improving QE substantially across the visible range [101, 116]. Silicon detectors can reach QE approaching ≈90% in the visible and remain sensitive up to ≈1100 nm, well suited to visible‐NIR UPE; detection at longer wavelengths requires alternative materials and is typically more noise‐limited. During an exposure, photoelectrons accumulate in each pixel; after exposure, charges are shifted row by row to a readout node and converted to voltage, introducing readout noise on the order of a few electrons root mean square (RMS).

For UPE, two noise sources dominate: dark current (thermally generated electrons during exposure) and read noise (added during readout). In an uncooled CCD, dark current can be many electrons per pixel per second, completely overwhelming UPE level signals. Even with cooling, very long exposures accumulate dark signal and hot pixels whose shot noise grows as $\sqrt{t}$ where t is the integration time, whereas read noise imposes a floor when many short exposures are summed [101].

Deep‐cooled, back‐illuminated CCDs with dark current below 10^−3^ e^−^ pixel^−1^ s^−1^ can image strongly stimulated samples and, with integration times of tens of minutes and pixel binning, spontaneous emission from large or bright emitters such as yeast cultures, plant leaves and human skin [101, 102, 103, 104, 105]. For spontaneous UPE from small samples or where temporal resolution matters see Section 2.2. Standard CCDs are generally impractical as primary detectors.

#### 4.1.4 Electron‐Multiplying CCDs

EMCCDs introduce an on‐chip gain register that stochastically multiplies the signal before the readout node, effectively suppressing the impact of readout noise [113]. This enables single‐photon level imaging at each pixel, especially when combined with deep cooling to minimise dark current. In practice, EMCCDs can resolve individual photon events in short exposures and accumulate images of ultra‐weak emission over minutes to hours.

Their advantages and limitations for UPE are summarised in Table 4.

Cryogenically cooled EMCCDs further suppress dark current, enabling multi‐minute to hour‐long exposures in which almost all accumulated charge arises from photons, but they do not eliminate clock‐induced charge or shot noise which is largely temperature‐independent and can increase when higher clock amplitudes are needed at low temperature, nor the excess noise of the multiplication process [117, 118, 119]. For UPE, EMCCDs are particularly powerful when spatial patterning of emission (e.g., across tissues, cell monolayers or plant leaves) is of primary interest and integration times of many seconds to minutes are acceptable.

#### 4.1.5 Scientific CMOS Cameras

sCMOS cameras use per pixel amplifiers and parallel readout, achieving low read noise (typically 1‐2 e^−^ RMS), high frame rates and large sensor formats [94, 110, 114, 120]. Modern sCMOS devices can reach QE ∼60%–80% and offer wide dynamic range due to dual‐gain architectures and high‐resolution ADCs [19].

Their advantages and limitations for UPE are summarised in Table 4.

In summary, standard CCDs are rarely adequate for spontaneous UPE detection; EMCCDs are preferred for ultra‐weak spatial imaging, whereas sCMOS is useful for faster, higher flux scenarios (e.g., stimulated UPE or bioluminescence) where temporal resolution and field of view are prioritised over ultimate sensitivity.

### 4.2 Prospective and Emerging Detectors

Table 5 summarises the prospective and emerging detector technologies discussed in this section, together with their current status for biological UPE, and Table 6 their advantages and limitations. Most have excellent single‐photon performance in physics and quantum optics. Currently, there is little or no published validation in biological UPE measurement.

**TABLE 5:** Prospective/Emerging Detector technologies with potential UPE/Biophoton applications.

Detector	Peak QE	Spectral range	Dark noise (typical)	Operating temp	UPE track record
SPAD/SPAD arrays	Up to ∼70% (si, visible); <30% (InGaAs, NIR)	Si: ∼400−1000 nm; InGaAs: ∼900−1700 nm	Si: tens–hundreds cps; InGaAs: tens of thousands cps	Room temp to mild cooling	Some biophotonics use (fluorescence lifetime imaging (FLIM), etc.); direct biological UPE application limited. NIR dark counts are a serious obstacle.
SNSPD (superconducting nanowire single photon detector)	Up to ∼93%–98%	Broad: UV to mid‐IR	Extremely low: $<10^{-3}$ to ∼1 cps (filtered)	<2.5 K (cryogenic)	No established biological UPE track record. Cryogenic requirement and fibre coupling make whole‐sample biological imaging impractical at present.
TES (transition‐edge sensor)	Up to ∼95%	Broad; energy‐resolving	Very low	∼100 mK (sub‐kelvin)	Energy‐resolving (spectral) capability is attractive in principle; no established biological UPE use. Extreme cooling and slow response are barriers.
MKID (microwave kinetic inductance detector)	Moderate–high	Broad; energy‐resolving	Low	Sub‐kelvin	Astronomy‐developed [121]; energy + arrival‐time resolution intriguing for UPE in principle, but no biological validation.
Plasmonic/hot‐electron & WGM (whispering gallery mode) nanophotonic sensors	Device‐dependent	Device‐dependent	Device‐dependent	Often room temp	Early‐stage; theoretical/proof‐of‐concept. No demonstrated biological UPE measurement. Speculative for this application.

**TABLE 6:** Advantages and limitations of prospective and emerging detector technologies for UPE measurement. Specifications and current biological status are summarised in Table 5.

Detector	Advantages for UPE	Limitations for UPE
SPAD	Single‐photon timing with tens‐of‐picosecond jitter, enabling TCSPC, correlation and lifetime analysis. Visible QE ∼50%–70% [99]. Low‐voltage and compact; fibre‐coupled or on‐chip. Available as single devices and as linear and 2D arrays [122].	Avalanche light emission can cause optical crosstalk or spurious signals [99]. Dark counts (tens to hundreds s^−1^, si) exceed the best PMTs, and afterpulsing adds correlated false events. Dead time (tens of ns) limits count rate. Array fill factor reduces effective QE; pixel variation and crosstalk complicate quantitative imaging.
SNSPD	Dark counts ≪1 s^−1^. Detection efficiency ≥ 90% in selected visible and NIR bands. Timing jitter of tens of picoseconds [123, 124, 125, 126].	Operation at 2–4 K in closed‐cycle cryocoolers, with vibration, electromagnetic and thermal management. Single pixels or small arrays (∼10‐10^3^ pixels), typically fibre‐coupled: Point measurements only. Integration with biological samples is complex, specialised and costly.
TES	Near‐unity QE over a broad range (e.g. 400–2800 nm), with per‐photon energy and hence wavelength, resolution [123].	Operation at ∼100 mK, requiring dilution or adiabatic demagnetisation refrigerators. Slow response and small pixel counts. Same integration challenges as SNSPDs.
MKID	Single‐photon sensitivity with energy resolution, enabling low‐resolution spectroscopy. Negligible intrinsic dark current; readout limited by electronics [4]. Frequency multiplexing allows thousands of pixels on one feedline.	Millikelvin to few‐kelvin operation, specialised RF electronics and magnetic and microphonic shielding. Resonator ring‐down limits per‐pixel count rate. Research‐prototype status for biological UPE.
WGM resonator	High Q ($10^{6}$‐$10^{8}$) enhances the intracavity field, potentially to UPE‐level sensitivity. Mode selection targets specific wavelength bands. Room‐temperature, compact and compatible with microfluidics and tissue contact [127].	Does not count photons: Analogue resonance shifts need modelling and calibration to yield flux or spectra. Small mode volume; larger samples need arrays or scanning. Sensitive to thermal drift, vibration and refractive‐index fluctuation. Requires tuneable‐laser readout.
Plasmonic (nanogold)	Room‐temperature, potentially single‐photon detection via hot‐carrier photocurrents. Near‐field coupling, with potential subcellular resolution in arrays. Femtosecond to picosecond response, limited by electronics. CMOS‐integrable [128].	Low external QE (internal efficiencies up to ∼20% in idealised devices). Narrowband, with a small capture cross‐section, so broadband or wide‐area measurement needs large arrays. Nanoscale variability requires per‐sensor calibration.

#### 4.2.1 Single‐Photon Avalanche Diodes and Time‐Resolved Approaches

SPADs are solid‐state devices operated in Geiger mode: a reverse‐biased p‐n junction is held above breakdown so that the absorption of a single photon can trigger a self‐sustaining avalanche, read out as a current pulse. The avalanche is then quenched electronically to reset the device [122]. SPADs achieve single‐photon sensitivity with timing jitter down to tens of picoseconds and can be fabricated as compact single detectors or as arrays for imaging.

Overall, SPADs bridge a niche between PMTs and EMCCDs: they enable high‐precision timing and photon counting with solid‐state convenience, at the cost of higher dark noise and more complex artefacts. For UPE studies focussed on temporal structure (e.g., burst kinetics, DL decay profiles) rather than spatial mapping, SPADs are a promising and rapidly evolving option.

#### 4.2.2 Cryogenic Single‐Photon Detectors

A class of cryogenic detectors superconducting nanowire single‐photon detectors (SNSPDs), transition‐edge sensors (TES) and microwave kinetic inductance detectors (MKIDs) offer essentially background‐free single‐photon sensitivity with excellent timing and, in some cases, energy resolution. They are natural candidates for pushing UPE detection towards its physical limits, but at the cost of substantial complexity.

#### 4.2.3 SNSPDs and TES

SNSPDs consist of a narrow superconducting wire biased close to its critical current and cooled to a few kelvin. Absorption of a photon locally breaks superconductivity, forming a resistive hotspot that diverts current and generates a voltage pulse [123, 124, 125, 126].

TES detectors operate as superconducting microcalorimeters biased in the sharp transition between superconducting and normal states. Each absorbed photon heats the sensor slightly, changing its resistance; this can yield near unity QE over a broad spectral range (e.g., 400–2800 nm) and also provide energy (wavelength) resolution per photon [123].

For UPE, both are ideal point detectors in sensitivity and noise; their practical limitations, cryogenic, small pixel counts and integration cost, are summarised in Table 6.

In practice, cryogenic detectors are best suited for proof of principle measurements that set upper bounds on spontaneous UPE under idealised conditions, or for spectrally/temporally resolved studies of stimulated emission by specialised teams. For routine biological work, their complexity currently limits widespread adoption.

#### 4.2.4 Microwave Kinetic Inductance Detectors

MKIDs are superconducting resonators whose resonance frequency and quality factor change when Cooper pairs are broken by photon absorption [4, 129]. An MKID array consists of many resonators with distinct microwave frequencies, all coupled to a common readout line, allowing frequency division multiplexed detection of thousands of pixels [121, 129].

In principle, MKIDs could provide near background‐free, spectrally resolved UPE imaging [121], directly constraining the spectral distributions predicted in Section 3.2 and resolving the chemiexcitationemitters of Section Section 2. In practice, they are likely to complement PMTs or EMCCDs.

#### 4.2.5 Emerging Nanophotonic and Plasmonic Sensors

Beyond conventional and cryogenic photon counters, several emerging platforms aim to enhance or transduce UPE via nanophotonic and plasmonic resonances. Three representative classes are whispering gallery mode (WGM) microresonators, nanoplasmonic (nanogold) wire‐based sensors and related hybrid structures [4, 127, 128, 130, 131, 132].

#### 4.2.6 Whispering Gallery Mode Resonators

Whispering gallery mode (WGM) sensors use dielectric microcavities (microspheres, microtoroids, micro‐rings) that support high‐Q optical resonances via total internal reflection. Photons circulating in the resonator build up an enhanced intracavity field; small changes in refractive index, absorption or scattering near the surface shift the resonance frequency or linewidth [4].

For UPE, a WGM resonator placed in close proximity to cells can, collect and enhance otherwise undetectable photons. WGM sensors, transduce UPE into analogue resonance shifts, with the consequences summarised in Table 6.

WGM resonators are therefore best used as highly sensitive, spectrally selective probes of local UPE or related refractive index changes, complementary to more direct photon counting methods [127].

#### 4.2.7 Emerging Nanosensors: Beyond Direct Photon Counting

##### 4.2.7.1 WGM Microcavities as Photon Transducers and Precursor‐State Sensors

WGM resonators can complement direct UPE photon counting in two conceptually distinct ways.

- Photon Transduction. In principle, a single absorbed photon can be converted into a measurable molecular event (e.g., a conformational/photocycle transition in a high quantum yield photoreceptor) that is then read out by a WGM sensor. This is an indirect photon‐detection paradigm: the biological receptor performs the photon‐to‐state conversion, whereas the cavity provides high‐sensitivity readout.
- Precursor‐State Sensing. Chemiexcitation models of UPE predict short‐lived, high‐energy intermediates (radical/absorbing states) that precede photon emission. WGM optoplasmonic sensing can detect molecular absorption and energetic state changes at the single‐molecule level; thermo‐optoplasmonic (TOP) sensing, for example, reports anomalous absorption and intensity‐dependent resonance shifts consistent with heat generation from absorbed near‐IR cavity fields. Such approaches can map the presence and kinetics of reactive intermediates and radical states, thereby constraining mechanistic pathways upstream of photon emission.

Crucially, precursor sensing provides mechanistic constraints that become most powerful when paired with simultaneous photon‐counting measurements.

#### 4.2.8 NV Centres and Other Quantum Sensors

Nitrogen‐vacancy (NV) centres in diamond and related quantum sensors offer another route to detect paramagnetic species (unpaired‐electron states) at high sensitivity, potentially enabling radical detection in regimes inaccessible to bulk spectroscopy. As with WGM precursor‐state sensing, these approaches are complementary to UPE detection: they probe radical generation and decay, not photons directly.

#### 4.2.9 Nanogold Wire‐Based Plasmonic Sensors

Gold nanowires, rods and related structures support localised surface plasmon resonances: collective oscillations of conduction electrons that strongly enhance local electromagnetic fields [128, 130]. When coupled to rectifying junctions (Schottky barriers, metal‐insulator‐metal diodes), plasmon decay can generate hot carriers that produce measurable photocurrents.

Their advantages and limitations for UPE are summarised in Table 6.

Conceptually, nanogold and WGM approaches share an emphasis on resonant enhancement and near‐field coupling, and may ultimately be combined (e.g., plasmonic structures within dielectric resonators) [133, 134, 135]. At present, they represent promising but still emerging complements to established detectors.

### 4.3 Practical Recommendations for UPE Measurements

Across detector technologies, the fundamental requirement for UPE experiments is that the expected signal count S from the sample over an integration time T is distinguishable from the combined dark and background counts, $N_{\text{dark}}$, with $S/\sqrt{S+N_{\text{dark}}}\geq 1$ for minimally acceptable SNR. Spontaneous UPE from well‐coupled oxidative phosphorylation (Section 2.2) is expected to be extremely weak (Section 3.2), so this condition is very hard to satisfy in practice.

Taking a pragmatic view, this section has been summed up as follows.

- Spontaneous UPE. For attempts to detect spontaneous UPE from metabolically normal systems, the lowest possible dark counts are essential. Because the emitters are the same chemiexcited species as under stress, spanning the UVA to the red, low‐noise visible‐range PMTs remain the first choice, with cryogenic SNSPD, TES or MKID systems as benchmarks. Imaging detectors (EMCCD, sCMOS) will usually be limited to stimulated or higher‐flux regimes.
- Stimulated UPE. In regimes where emission is boosted by strong stressors, visible range UPE can be orders of magnitude brighter. EMCCD and sCMOS cameras become viable, enabling spatial mapping, whereas PMTs and SPADs remain optimal for high‐fidelity time series and photon counting kinetics (Section 1.3).

More specifically, for typical biological UPE experiments.

- Point counting versus imaging. If total photon flux, temporal dynamics or spectral signatures at one location, are required, a PMT, SPAD or cryogenic detector coupled via fibre or confocal optics is preferred. Spatial distribution information (e.g., across tissues or plant leaves), an EMCCD (for ultra‐weak, slowly varying signals) or sCMOS (for brighter, more dynamic signals) is more appropriate.
- Timescale. Long‐term monitoring (minutes‐hours) requires detectors with stable baselines, low dark noise and robust shielding (PMTs, EMCCDs, cryogenic detectors). High‐frame‐rate studies of rapid transients (e.g., triggered bursts, DL decays) may require SPADs or sCMOS detectors.
- Spectral regime. In both regimes, chemiexcitation emitters span ∼380–750 nm, with the ^1^O_2_ monomol band at 1270 nm (Section 2.1); visible‐range detectors cover most of this. NIR sensitivity is needed for the 1270 nm band, where the sample's thermal emission requires differential acquisition (Section 4.3.1). Direct ETC emission, beyond ∼2000 nm, lies beneath that thermal emission and cannot be isolated by any detector (Section 3.3)
- System‐level noise control. Regardless of detector choice, careful shielding, dark/background subtraction and artefact rejection are mandatory; these are discussed in detail below.
- Physiological positive controls. In addition to mechanistic insight, oxygen delivery perturbation via arterial occlusion (± reperfusion) provides a convenient end‐to‐end instrumentation control for UPE measurements: it is noninvasive, repeatable within subject and has been reported to induce a decrease in UPE during occlusion with a postrelease change during reperfusion [58] (Section 2.3). This can therefore be used to validate low‐photon detector performance (baseline stability, stray light rejection, drift handling and analysis robustness), ideally alongside simultaneous haemodynamic/oxygenation monitoring (e.g., HbO, HbR) to interpret absorption/scattering changes that accompany the perturbation.

In practice, many advanced setups combine multiple detector types: for example, a PMT or SPAD channel for high‐fidelity photon counting and kinetics, an EMCCD for spatial mapping over longer integrations, and, in exploratory work, a cryogenic or nanophotonic sensor for reference measurements or spectral benchmarking. Such hybrid systems recognise that no single detector can simultaneously optimise all desired attributes (sensitivity, spatial/temporal resolution, spectral coverage, practicality and cost), especially at the extreme low light levels relevant to spontaneous UPE.

Taken together with the energetic constraints of Section 3, these practical considerations reinforce the central message of this review: under realistic conditions, spontaneous mitochondrial UPE is expected to be an extremely weak by‐product of oxidative metabolism, often beyond the reach of standard detectors. Robust UPE signals measured measured in many experiments are far more plausibly interpreted as arising from stimulated, high ROS, high damage regimes and experimental designs should be constructed and analysed with this two‐regime framework in mind.

#### 4.3.1 Noise, Artefacts and Calibration

Dark‐count correction. Dark counts thermally generated events with no incident photon are the dominant background; for photon‐counting UPE at low flux. They must be measured under conditions identical to signal acquisition (same temperature, integration time, gain, and shutter state) and subtracted, with the Poisson uncertainty of the dark measurement propagated into the reported value. Because dark rate depends steeply on temperature, dark frames should bracket or interleave the measurement and detector temperature be logged throughout; a single up‐front dark calibration is inadequate for hour‐long integrations.

Cosmic‐ray and spurious‐event artefacts. Over the long integrations UPE requires, cosmic rays, environmental radioactivity, and detector afterpulsing produce rare high‐amplitude events that can masquerade as bursts. They can be distinguished from genuine signal by their statistics non‐Poisson, clustered, or of anomalous pulse height. These are removed by amplitude thresholding, spatial filtering (cosmic‐ray hits deposit in few pixels on imaging sensors), or coincidence rejection. For claims of transient UPE especially, they must be explicitly characterised and removed.

Ambient‐IR and thermal background. Every object at room or physiological temperature emits thermal radiation. The dominant source in the NIR is the sample itself (Section 3.3), which cannot be cooled. At 310 K, thermal photon exitance per 100 nm band is $∼10^{-2}$ photons s^−1^ cm^−2^ at 800 nm, ∼3 at 900 nm, $∼2\times 10^{2}$ at 1000 nm and $∼10^{6}$ at 1270 nm [86, 87] (blackbody upper bounds; thin aqueous samples, which absorb weakly below ∼1300 nm, emit less [136]). Against reported UPE intensities of $10^{-1}$‐$10^{2}$ photons s^−1^ cm^−2^, the thermal floor therefore crosses UPE levels between ∼850 and 1000 nm. Below this crossover, absolute measurement is limited by detector and stray‐light backgrounds; above it, only differential or modulated acquisition against a stable baseline can isolate UPE, and in practice only up to ∼1200–1300 nm. Because thermal flux at 1000 nm changes by ∼15% per kelvin, sample temperature must be stable at the millikelvin to tens‐of‐millikelvin level for such measurements. Warm surfaces, imperfect light‐tight seals and the detector housing add to this background, so light‐tight, thermally controlled enclosures, cooled optics and NIR‐specific dark controls are required: a shielding regime validated only in the visible does not guarantee a clean NIR measurement.

Photon‐counting statistics. At UPE levels most detection intervals contain zero or one photon, so signal and background follow Poisson statistics. What matters is whether the signal excess is statistically significant against the combined dark‐plus‐background variance (the $S/\sqrt{\left(S+N_{\mathrm{dark}}\right)}$ criterion above), which sets minimum integration times and total‐count requirements; the raw mean count alone is uninformative at these levels. For imaging detectors, per‐pixel Poisson noise plus read and multiplication noise must be treated explicitly, with spatial or temporal binning traded against resolution to reach significance.

Inter‐laboratory calibration. Intensities in photons$\cdot s^{-1}\cdot cm^{-2}$ (or per cell) depend on QE, collection geometry (solid angle, NA), optical throughput and sample area quantities that differ widely between setups. Absolute calibration against a traceable low‐flux source, together with reporting of these parameters, is required; allowing, count rates from different laboratories to be comparable. Calibration to absolute photon flux, with stated collection efficiency and geometry, should be a minimum for quantitative claims.

Spectral‐response correction. Every detector has a wavelength‐dependent QE, so a raw spectrum is the convolution of the true emission with the detector response. A silicon detector's falling sensitivity beyond ∼800 nm suppresses the long‐wavelength chemiexcitation components, the red tail of pigment emission (Section 3.2) and the ^1^O_2_ monomol band at 1270 nm, which can manufacture a spurious blue bias in the recorded spectrum. Reported spectra should be corrected for the combined spectral response of detector, filters and optics, with the effective sensitivity window stated so that an apparent absence of NIR emission is not an artefact of the instrument.

## 5 Applications Within a Two‐Regime Framework

As a noninvasive optical marker reflecting the level of oxidative activity and damage within a system, UPE has been explored for applications in medicine, agriculture, food science and fundamental biology. In almost all of these cases, however, the experimentally accessible signals correspond to the stimulated/high‐stress regime defined in Sections 2.3 and 3.2, rather than to the extremely weak spontaneous emission expected from well‐coupled oxidative phosphorylation. Below, we briefly summarise the major application domains and interpret their reported intensities and spectra in light of the energy constraints developed in Section 3.

### 5.1 Medical and Health Applications

Most reported ‘medical UPE’ studies probe tissues in which oxidative metabolism is strongly perturbed: malignant growth, ischemia‐reperfusion, inflammation or intense functional activation. The measured signals therefore predominantly sample the stimulated/high ROS regime, where lipid peroxidation and other damage processes generate visible chemiluminescence on top of any spontaneous mitochondrial emission [14, 16, 17, 107, 137].

#### 5.1.1 Cancer Detection and Imaging

Tumour cells often exhibit altered metabolism, elevated ROS production and extensive membrane remodelling. Several groups have reported that UPE from tumour bearing tissues or xenografts is significantly higher than from surrounding normal tissue, and can track tumour burden [14, 16, 17]. Using ultra‐sensitive PMT and EMCCD systems, Takeda et al. (2004) [137] demonstrated that photon emission from implanted tumours in rodents increases with tumour size and viability, and decreases following effective chemotherapy, suggesting a link between emission intensity, proliferative activity and oxidative stress. Although such approaches are sometimes framed as detecting ‘intrinsic biophotons’, their sensitivity relies on the fact that malignant tissue is metabolically dysregulated and experiences chronic or treatment‐induced oxidative damage.

#### 5.1.2 Brain Activity and Neurodegeneration

The brain has one of the highest metabolic activities of any organ and an intense producer and consumer of ROS. UPE has been measured from brain slices, in vivo cortical surfaces and isolated neural tissue, with reports of correlations between photon counts, electrophysiological activity and pathological states [18, 74, 138]. Experiments on anaesthetised animals indicate that UPE patterns change between rest and stimulation, and may show rhythmic components that echo underlying neuronal oscillations [18, 107]. In models of neurodegeneration, increased emission has been associated with mitochondrial dysfunction, protein aggregation and enhanced lipid peroxidation, consistent with a shift into the stimulated regime [138]. The detectable signal appears to be dominated by ROS linked chemiluminescence rather than by the near invisible spontaneous glow of healthy mitochondria.

Given that some authors have proposed photonic propagation mechanisms in neural structures, we summarise these ideas conservatively here, focussing on constraints and falsifiable tests rather than functional claims.

#### 5.1.3 Noninvasive Metabolic Monitoring

A related line of work uses UPE and closely related low‐level chemiluminescence to track metabolic and redox status in accessible tissues such as skin, retina and mucosa [19, 26, 107, 139, 140, 141]. EMCCD and sCMOS cameras have been used to map spatial patterns of emission across tissue surfaces [25, 26], sometimes in conjunction with functional perturbations (e.g., ischemia‐reperfusion, pharmacological challenges) or with co‐registered fluorescence/autofluorescence imaging. These studies report that regions with higher oxidative load or impaired antioxidant capacity tend to display stronger UPE, and that temporal changes in emission can follow metabolic interventions. Such approaches are promising as noninvasive reporters of oxidative insult and redox imbalance, especially when combined with complementary imaging modalities [19, 107, 139, 140, 141].

Viewed through the quantitative constraints of Section 3, the intensities and visible spectra reported in most medical/health applications are far too strong, and too rich in blue‐green components, to be explained by spontaneous ETC linked emission. Instead, they are naturally understood as manifestations of the stimulated/high damage regime: chemiexcitation from ROS bursts, lipid and protein oxidation and DL following UV or other energetic priming. Clinical or preclinical UPE imaging in oncology and neurology should therefore be interpreted primarily as a readout of oxidative stress and tissue damage, not as a direct optical window onto routine mitochondrial ATP production.

### 5.2 Agricultural Applications (Plant Health and Seed Quality)

Plant and seed studies likewise almost always operate in a stimulated regime. Plants are exposed to strong abiotic (light, temperature, drought, salinity) or biotic (pathogen, pest) stressors and seeds are probed at different stages of ageing or germination where redox balance is strongly perturbed. Under these conditions, UPE tracks the extent and dynamics of oxidative processes in chloroplasts, mitochondria and membranes [18, 55, 56].

#### 5.2.1 Stress and Disease Monitoring in Plants

The abiotic physiological stress response in plants is closely tied to ROS production in chloroplasts and mitochondria, and UPE has been used as a noninvasive marker of this response. Prasad et al. (2020) [18] showed that plant leaves exposed to drought, low temperature or mechanical damage emit significantly more photons than unstressed controls, with emission increases of several orders of magnitude in severe conditions [55, 56]. Biotic stress, including pathogen infection and herbivory, similarly elevates UPE, often with spatial patterns that reveal the spread of localised responses before visible symptoms appear. These observations fit naturally with a picture in which oxidative bursts and lipid peroxidation in stressed tissues dominate the measurable emission.

#### 5.2.2 Seed Quality and Germination

Seed viability and vigour are intimately linked to redox homeostasis and the integrity of membranes and macromolecules. UPE measurements on dry and imbibed seeds have been proposed as a rapid, nondestructive way to assess seed quality and predict germination success [18, 142]. Griffo et al. (2024) [142] carried out studies comparing freshly harvested, stored and artificially aged seeds report that photon emission intensity and decay kinetics correlate with classical viability metrics, such as germination percentage and seedling vigour [67]. High emission seeds often show evidence of advanced oxidative damage and poor subsequent performance, whereas low emission seeds tend to be healthier. In this context UPE functions as a proxy for accumulated oxidative stress and the balance between protective and damaging processes during storage and early germination.

In light of Section 3, the relatively strong visible UPE and its sensitivity to acute stressors in plant and seed experiments argue again for a stimulated/high ROS interpretation. The observed bands are consistent with chlorophyll related chemiluminescence and ROS signatures (triplet carbonyls, ^1^O_2_) rather than with spontaneous mitochondrial emission. Although this does not diminish the value of UPE as an agronomic or seed quality metric, it reinforces that these applications quantify oxidative stress and damage, not steady state photosynthetic or respiratory photon leakage.

### 5.3 Food Quality and Safety

Food quality and safety applications of UPE are, by design, focussed on oxidatively stressed or degrading material. Here the measured emission is generated largely by lipid and protein oxidation, microbial spoilage and other deterioration processes, therefore within the stimulated regime [14, 16, 17, 19].

Several studies have suggested that UPE can be used to assess freshness and oxidative status of fruits, vegetables, meats and oils. For example, Nematollahi et al. (2020) [17] showed that stored meat and fish samples exhibit higher UPE intensities than fresh samples, with emission increasing over time in parallel with markers of lipid peroxidation and microbial growth. Similar trends have been reported for plant‐based foods and edible oils, where photon counts and spectral shapes change systematically as oxidative rancidity develops [14, 16]. These correlations have led to the proposal that UPE could underpin noncontact quality control systems in the food industry, enabling real‐time, nondestructive assessment of spoilage and shelf life [19].

Beyond quality, there is interest in using UPE to monitor food safety in relation to contamination, mycotoxins and storage conditions [17]. Since many safety‐relevant processes (microbial proliferation, toxin production, oxidative stress under improper storage) involve intense ROS chemistry, they can induce detectable changes in photon emission. The ability to interrogate packaged or delicate products without physical sampling is particularly attractive for automated inspection lines.

From the viewpoint of Section 3, the food‐related UPE signals are entirely consistent with a high damage, ROS driven origin. Intensities are orders of magnitude higher than any plausible spontaneous emission from intact, healthy tissues and spectra often span the visible range associated with lipid peroxidation and ^1^O_2_ emission. These applications therefore exemplify how UPE can be fruitfully exploited as a marker of oxidative deterioration, while at the same time illustrating that such strong visible signals cannot be regarded as representative of spontaneous metabolic photon production.

### 5.4 Dermatology and Skin Care (Sun Cream Applications)

Skin‐based UPE studies almost universally involve deliberate photic or chemical stimulation. Human or animal skin is exposed to controlled doses of UVA/UVB or visible light, sometimes combined with topical photosensitisers, antioxidants or sunscreens, and the resulting emission is monitored over seconds to hours [51, 52, 64, 65, 137]. These experiments probe the stimulated regime in which UV induced ROS, lipid peroxidation and protein oxidation in the epidermis and dermis generate substantial chemiluminescence.

Work by Ou‐Yang et al. (2004) [52] demonstrated that UVA irradiation of human skin produces a pronounced increase in UPE, with a broad visible spectrum that decays over time and is sensitive to antioxidant status. Subsequent studies refined this approach, using PMTs and EMCCDs to characterise dose response relationships, kinetics and spectral changes in UV exposed skin [52, 137]. Application of sunscreens or antioxidant formulations typically reduces the amplitude of UV induced UPE, accelerates its decay or shifts its spectral composition, reflecting reduced ROS generation and improved quenching of excited intermediates [64, 65]. These properties have led to proposals that UPE measurements could serve as functional assays for sunscreen efficacy, oxidative photodamage and the protective effects of cosmetic formulations.

Skin is optically accessible and relatively homogeneous at the macroscopic scale, it is an attractive testbed for instrument development and for exploring DL phenomena. Nonetheless, the absolute intensities and clear dependence on UV dose and antioxidant treatment align closely with the stimulated/high‐stress regime described in Section 2.3. The spectra are dominated by visible bands characteristic of lipid and protein oxidation products, at intensities far above the spontaneous baseline of Section 3.2. Dermatological UPE measurements are therefore best interpreted as sensitive optical reporters of UV induced oxidative stress and its mitigation, rather than as direct measurements of routine skin metabolism.

### 5.5 Fundamental Biological Research

A final application domain comprises more speculative or exploratory uses of UPE in fundamental biology: as a probe of metabolic organisation, as a candidate channel for intercellular communication and as a possible manifestation of quantum or microtubule‐based photonic processes [4, 8, 143]. Much of this work either operates in the stimulated regime or extrapolates from high‐stress experiments to hypotheses about spontaneous conditions.

Several studies have explored whether UPE can mediate or report on long‐range signalling between cells or tissues. Following the early experiments of Gurwitsch on ‘mitogenetic rays’ from dividing onion roots [13], modern re‐investigations have used improved optics and photon counting detectors to test whether photon emission from one cell population can influence proliferation or gene expression in a spatially separated partner [69, 70, 144]. Some groups report modest but statistically significant bystander like effects that appear to depend on optical coupling, whereas others fail to reproduce such findings. Importantly, many of these experiments involve highly proliferative, stressed or irradiated tissues that emit at comparatively high levels, consistent with the stimulated regime; whether any analogous photonic coupling exists at spontaneous emission levels remains unresolved and, in light of the low photon budgets in Section 3, is energetically challenging.

Hypotheses have also been advanced in plant systems germination and root system development which could be influenced by endogenous photon fields, with UPE patterns reflecting or modulating the progression of growth [67]. On a smaller scale, microtubules have been proposed to act as nano‐optical waveguides, carrying biophotons over cellular or subcellular distances and potentially supporting quantum coherent processes relevant to neural computation [8, 73, 143]. In such models, the high refractive index contrast between microtubules and cytosol is invoked to confine light, and quantum electrodynamics (QED) based calculations suggest the possibility of bound photonic modes. However, maintaining guided, coherent photonic signals over biologically meaningful distances in the warm, scattering, absorbing environment of the cytoplasm is likely to be extremely difficult: thermal noise, absorption and structural disorder all act to decoherent and attenuate any putative waveguide modes on short length scales.

Subcellular ‘waveguiding’ hypotheses (myelinated axons, microtubules). What would it take to test? The possibility that endogenously generated photons might propagate as guided modes in neural structures has been explored largely in theoretical/modelling work for myelinated axons (dielectric waveguide hypothesis) [75], alongside more speculative proposals involving microtubules. In parallel, ultraweak photon emission has been experimentally reported from neural preparations and/or active nerve tissue (i.e., evidence for emission, not guidance) [145, 146, 147]. For visible wavelengths, several constraints motivate a conservative framing. In particular, microtubule diameters (∼25 nm) imply that classical dielectric guidance of visible light is not straightforward; in tissue, additional losses arise from refractive‐index heterogeneity, scattering/absorption and bends/discontinuities. Critically, even if photons exist, a biologically plausible launching/coupling mechanism and a background‐free readout must be demonstrated. A negative experimental programme would therefore separate emission from propagation.

1. Establish stimulus‐locked emission above artefact controls. Demonstrate repeatable, stimulus‐locked photon production with strict dark controls and confound monitoring (stray‐light rejection; electrode/electrochemistry luminescence; temperature; tribo‐/chemo‐luminescence; probe photochemistry), building on prior emission reports [145, 146, 147].
2. Test propagation independently by controlled optical injection. Inject calibrated light into isolated, well‐characterised myelinated fibres and map transmission versus distance (loss length), wavelength and geometry; then perturb the putative waveguide (local demyelination, index matching, micro‐lesions) and test for predicted changes [75].
3. Only then test ‘endogenous guidance’ signatures. Look for spatially separated, time‐locked correlations along an axon with distance‐dependent attenuation and modality‐specific signatures (e.g., spectral filtering/polarisation/mode‐dependent loss) that cannot be explained by local emission alone.

Until such propagation‐specific tests are available, ‘waveguiding’ is best treated as a hypothesis rather than an established biological signalling mechanism.

More conservative fundamental studies use UPE as a passive reporter of metabolic organisation and ROS dynamics, without invoking exotic communication channels. These include comparisons of emission patterns across cell types and developmental stages, or under perturbations that alter mitochondrial coupling, antioxidant capacity or membrane composition [4, 18]. Such work fits naturally into the two‐regime framework articulated in this review: spontaneous UPE provides a weak background from basal chemiexcitation, whereas transitions into high‐ROS states (e.g., during differentiation, apoptosis or disease) raise UPE intensity by orders of magnitude.

Reported red shifts in glutamate‐induced brain biophotonic activity across species, reaching ∼865 nm in human slices [148], cannot reflect direct ETC energetics, which Section 3.2 places beyond ∼2000 nm. The emission was recorded under strong glutamate stimulation, which has been argued to drive ROS production through NADPH oxidase and nitric oxide synthase activation [149], although the original authors dispute a free‐radical origin [150]. Differences in emitter composition or tissue optics are more plausible explanations than an energetic one, and the data carry no direct evidence of a signalling role.

The fundamental research literature illustrates both the conceptual appeal and the energetic constraints of ‘biophoton’ hypotheses. The strongest experimental signals supporting intercellular photonic effects arise under conditions where tissues are clearly in a stimulated/high damage regime, with emission driven by ROS bursts and membrane breakdown. Extrapolating these findings to spontaneous conditions must contend with the extremely limited photon budgets of Section 3 and with the absence of any direct ETC emission channel in the visible or NIR. Any future models of photonic signalling or microtubule‐based light transport will need to be compatible with these quantitative constraints and with the dominant role of conventional chemical and electrical signalling pathways in biological communication.

## 6 Outlook and Conclusions

The picture that emerges from the quantitative analysis in Section 3, the detector limits in Section 4, and the application survey in Section 5 is conceptually simple. Ultra‐weak photon emission is a generic consequence of oxidative chemistry, yet the overwhelming majority of respiratory free energy is channelled into proton‐pumping, ATP synthesis and heat. The tiny fraction that leaks into radiative channels is strongly constrained both energetically and spectrally.

In spontaneous, well‐coupled mitochondria, these constraints exclude direct ETC‐linked emission from the visible and NIR. By contrast, the robust visible UPE signals reported in many biological and clinical studies almost invariably arise when systems are driven into a stimulated, high ROS, high damage regime by UV/visible irradiation, ischemia‐reperfusion, toxins, senescence or intense activation. In this regime, lipid peroxidation, protein oxidation, DL and other ROS driven chemiexcitation pathways dominate the emission and naturally produce stronger, structured visible spectra.

This two‐regime view reframes many long‐standing biophoton claims. It emphasises that any such roles must operate under severe photon budget and spectral constraints, and must be compatible with the dominant role of chemical and electrical signalling. The challenge for future work is therefore not only to detect spontaneous UPE reliably, but to design experiments that can discriminate between high‐stress chemiluminescence and subtle spontaneous signals, and to test putative photonic functions against realistic energetic bounds.

### 6.1 Key Constraints and What They Rule Out

Three constraints stand out. First, the energy budget of oxidative phosphorylation limits how many photons can plausibly be emitted per electron transferred. In well‐coupled mitochondria, most of the free energy from NADH and FADH_2_ is committed to the proton motive force and ATP synthesis; only a modest residue is dissipated at the terminal cytochrome c → O_2_ step, and only a minute subset of that can appear as photons. Spontaneous UPE must therefore correspond to an exceedingly small fraction of respiratory turnovers.

Second, the spectrum of any direct ETC‐linked emission is fixed by the size of individual redox steps, not by their sum. Per electron, the largest step, cytochrome c → O_2_, corresponds to ∼2180 nm, and after proton‐pumping at Complex IV the residual lies beyond ∼3000 nm (Section 3.2), where the sample's own thermal emission exceeds reported UPE by more than ten orders of magnitude (Section 3.3). Routine ETC activity cannot generate visible photons under any coupling condition; blue or green emission requires the concentration of energy into a single excited state that only secondary chemiexcitation provides.

Third, detector performance and background conditions impose hard limits on what can be measured. Even the best room‐temperature photon counters have finite dark counts, stray‐light sensitivity and thermal backgrounds that dominate at very low fluxes, and imaging detectors (EMCCD, sCMOS) carry additional penalties in dark current, read noise and fixed‐pattern noise (Section 4.3). In intact tissue, scattering and absorption further attenuate and spectrally distort any emergent spontaneous UPE.

These constraints call several biophoton hypotheses into question. Bright, coherent visible light arising directly from spontaneous mitochondrial metabolism in healthy tissue is energetically implausible, and high photon yields in the blue‐green region cannot be attributed to routine ETC turnover. Interpretations of strong visible UPE as a high‐capacity communication channel between healthy cells must also contend with the observation that such signals almost always coincide with high ROS.

None of this rules out ultra‐weak spontaneous emission from basal chemiexcitation, or more modest roles for UPE: as a sensitive marker of oxidative imbalance, for example, or as a weak modulator of redox‐sensitive pathways in very specific geometries. Any such proposal must be framed quantitatively and tested against the constrained photon budgets available.

### 6.2 Experimental and Analytical Priorities

Within this framework, priorities for future work fall into two broad categories: improving the physical characterisation of UPE (especially in the spontaneous regime), and tightening the link between photon emission and underlying oxidative processes.

On the physical side, there is a clear need for.

- Rigorous spectral measurements extending into the NIR. Most existing UPE spectra cover 400–800 nm, which captures most chemiexcitation emitters. Extension into 800–1700 nm, using InGaAs sensors, red/NIR‐optimised PMTs or cryogenic detectors, adds the ^1^O_2_ monomol band near 1270 nm and the red tail of pigment emission, and allows upper limits to be placed on any long‐wavelength component.
- Well‐calibrated photon budgets. Absolute photon fluxes, collection efficiencies and sample geometries must be quantified so that observed count rates can be compared directly with energy budget based expectations (Section 3). This includes careful characterisation of detector QE, dark counts, optical throughput and sample area/volume.
- Improved background control and subtraction. Dark frames, sample‐free controls, shielding from stray light and thermal emission, and robust treatment of rare artefacts (cosmic rays, afterpulsing, electronic spikes) are mandatory if UPE is to be distinguished from instrumental noise.
- Targeted use of advanced detectors. Cryogenic detectors (SNSPDs, TES, MKIDs) and optimised SPAD systems can set stringent upper bounds on spontaneous emission under idealised conditions. Strategic benchmark experiments with such devices can complement more routine PMT/EMCCD work.

On the biological and analytical side, key priorities include.

- Simultaneous ROS/UPE measurements. Whenever possible, UPE recordings should be accompanied by independent measures of ROS and oxidative damage (e.g., fluorescent probes, electron paramagnetic resonance (EPR), biochemical assays). Correlating photon emission with ROS markers can help discriminate spontaneous from stimulated regimes and clarify the contribution of specific chemiexcitation pathways.
- Explicit regime classification. Experiments should explicitly state whether samples are expected to be in a spontaneous, near homeostatic state or in a stimulated/high‐stress state (UV exposed, oxidant‐treated, ischemic, senescent). This regime labelling is as important for interpretation as the raw photon counts.
- Integration with metabolic and electrophysiological data. Oxygen consumption rates, ATP levels, membrane potential and, where relevant, electrophysiological measurements provide critical context for interpreting UPE as a marker of metabolism or excitability.
- Quantitative testing of signalling hypotheses. Putative photonic communication or microtubule waveguide models should be confronted with explicit calculations of expected photon numbers, attenuation lengths and SNR under realistic conditions. Experiments should be designed to falsify as well as to support such models.
- Standardisation and replication. Agreed protocols for sample preparation, dark adaptation, integration times, spectral bands and data analysis would facilitate cross laboratory comparisons. Sharing raw photon count time series and calibration data would enable independent reanalysis and meta‐analyses.

### 6.3 Implications for Future Biophoton Research

Within the two‐regime framework developed in this review, a consistent set of implications emerges. UPE should be understood primarily as a by‐product of oxidative metabolism. In the spontaneous regime its intensity is so low that it is unlikely to play any dominant, information‐rich signalling role in most tissues, although it does provide a conceptually direct, if practically challenging, optical readout of the balance between oxidative phosphorylation, ROS production and antioxidant defences.

Whenever robust UPE is observed, with intensities measurable by standard detectors over modest integration times, the system is almost certainly in a high‐ROS, high‐damage regime. UPE is then best interpreted as a marker of oxidative insult, membrane breakdown, DL following photic priming or related damage processes. This is a strength for applications rather than a weakness: medical, agricultural, food‐quality and dermatological studies can exploit UPE as a sensitive reporter of stress and deterioration.

Spontaneous biophoton signalling ideas are not ruled out, but they are strongly constrained and testable. Any model that invokes spontaneous UPE as a functional signal must operate within the photon budgets implied by the respiratory energy budget; must show that putative target systems can detect and respond to such weak signals against much stronger noise sources; and must show that photonic pathways add explanatory or functional power beyond conventional chemical and electrical signalling. Carefully controlled, quantitatively framed experiments using modern detectors, rigorous background control and simultaneous ROS and metabolic readouts can test these conditions. Whether any spontaneous photonic role survives this scrutiny is an open question.

#### 6.3.1 Recommended Reporting for UPE Experiments

To support quantitative interpretation and cross‐sectional‐study comparison, it is recommended that future UPE studies, as a minimum, report.

- Detector details: detector type (PMT, EMCCD, sCMOS, SPAD, SNSPD/TES/MKID, nanophotonic sensor), manufacturer/model, operating temperature, QE curve or at least the main sensitivity band.
- Noise and background: dark count rate or dark current (with temperature and integration time), read noise (for imaging sensors), clock‐induced charge (for EMCCDs) and a description of shielding and ambient light rejection.
- Spectral band and optics: filters, monochromators or gratings used; effective wavelength range; collection geometry (objective NA, fibre type, solid angle, sample area/volume).
- Acquisition parameters: integration time per frame, total acquisition time, binning, frame rate and any temporal gating or time‐correlated methods.
- Calibration and units: procedures for converting counts to photons (or at least normalised counts), including flat‐fielding and background subtraction; sample normalised units (e.g., photons s^−1^ cm^−2^, photons s^−1^ cell^−1^) where feasible.
- Biological regime description: clear description of sample type, metabolic/physiological state, stressors or treatments applied, antioxidant conditions and classification as spontaneous versus stimulated/high‐stress according to the framework of Section 2.

#### 6.3.2 Priorities for Future Work

Future experiments should consider.

- Systematic spontaneous‐regime studies using the lowest‐noise detectors available to characterise basal chemiexcitation, and its dependence on coupling and antioxidant state, in well‐characterised model systems (e.g., isolated mitochondria, yeast, cultured cells).
- Combined ROS/UPE imaging that co‐registers photon emission with ROS probes, mitochondrial potential indicators and structural markers, clarifying the spatial and temporal coupling between oxidative events and emission.
- Standardised stress protocols (UV doses, oxidant concentrations, ischemia‐reperfusion models) to map how UPE transitions from the spontaneous to stimulated regime across tissues and species.
- Spectrally resolved measurements that explicitly search for predicted features (^1^O_2_ bands at 634, 703 and 1270 nm, and the triplet‐carbonyl band near 430 nm) and distinguish them from autofluorescence and instrumental artefacts. Because dimol emission is second order in [^1^O_2_], the ratio of 634/703–430 nm emission should rise with ROS flux, providing a direct test of the two‐regime framework. At 1270 nm the sample emits ∼10^6^ photons s^−1^ cm^−2^ per 100 nm band at 310 K, so detection there requires differential or modulated acquisition against a thermally stabilised baseline.
- Mechanistic tests of signalling hypotheses that combine optical manipulation (filters, shutters, photonic barriers) with readouts of candidate biological responses, under conditions that keep systems as close to spontaneous as possible.

By grounding future biophoton research in rigorous energetics, carefully characterised detection and explicit regime labelling, the field can move beyond qualitative speculation towards quantitatively constrained, reproducible science. In doing so, UPE may find its most robust roles not as a mysterious new signalling modality, but as a sensitive optical reporter of oxidative metabolism and stress state.

## Author Contributions

**Samuel Crowther:** writing–original draft, writing–review and editing, funding acquisition. **Alice Attenborough:** writing–review and editing. **Frank Vollmer:** funding acquisition, project administration, supervision.

## Funding

This work was supported by the Leverhulme Trust Research Grant (RPG‐2024‐198).

## Conflicts of Interest

The authors declare no conflicts of interest.

## Data Availability

Data sharing not applicable to this article as no datasets were generated or analysed during the current study.
